# Trends in weight change patterns across life course among US adults, 1988–2018: population-based study

**DOI:** 10.1186/s12889-023-17137-x

**Published:** 2023-11-06

**Authors:** Xingxing Sun, Tingting Du

**Affiliations:** 1 Department of Anesthesiology, Hubei Key Laboratory of Geriatric Anesthesia and Perioperative Brain Health, and Wuhan Clinical Research Center for Geriatric Anesthesia, Tongji Hospital, Tongji Medical College, Huazhong University of Science and Technology, Wuhan, 430030 China; 2 Department of Endocrinology, Tongji Hospital, Tongji Medical College, Huazhong University of Science and Technology, Wuhan, 430030 China; 3 Branch of National Clinical Research Center for Metabolic Diseases, Wuhan, Hubei China

**Keywords:** Obesity, Weight change, Trend

## Abstract

**Background:**

To examine trends in weight change patterns from young adulthood through midlife to late adulthood and their sex and racial/ethnic disparities among US adults from 1988 to 2018.

**Methods:**

A total of 48,969 participants from the National Health and Nutrition Examination Survey 1988–1994 and 2001–2018 were included.

**Results:**

The age-adjusted prevalence of stable non-obesity between young adulthood and midlife declined significantly from 84.1% (95 CI, 82.9-85.3%) in 1988–1994 to 68.7% (67.1-70.2%) in 2013–2018, and between midlife and late adulthood from 71.2% (69.2-73.1%) to 52.4% (50.5-54.2%). The magnitude of increase in the prevalence of weight gain from young adulthood to midlife (from 10.8% [9.9-11.6%] in 1988–1994 to 21.2% [20-22.3%] in 2013–2018; *P* < 0.001 for trend) was greater than that from midlife to late adulthood (from 14.1% [12.9-15.3%] to 17.2% [16.2-18.1%]; *P* = 0.002 for trend). The magnitude of increase in the prevalence of stable obesity from young adulthood to midlife (from 3.9% [3.1-4.8%] in 1988–1994 to 9.2% [8.2-10.3%] in 2013–2018; *P* < 0.001 for trend) was smaller than that from midlife to late adulthood (from 11.2% [10.1-12.2%] to 24.8% [23.3-26.3%]; *P* < 0.001 for trend). The declining trends in the prevalence of stable non-obesity and increasing trends in the prevalence of weight gain and stable obesity from young adulthood through midlife to late adulthood were also observed for all sex and race/ethnicity subgroups. The magnitude of decrease in the prevalence of stable non-obesity, and the magnitude of increase in the prevalence of weight gain from young adulthood through midlife to late adulthood were greater in men than in women (all *P* for interaction < 0.01). Weight gain patterns for those aged ≥ 65 years were substantially different from the younger age groups.

**Conclusions:**

More young people born in later years are encountering obesity and accumulate greater obesity exposure across their lives than young people born in earlier years.

**Supplementary Information:**

The online version contains supplementary material available at 10.1186/s12889-023-17137-x.

## Background

The obesity epidemic is a major global public health concern. Many studies have examined trends in obesity to provide evidence to inform policy efforts and prevention programs [1–3]. However, most of these prior studies ignored the patterns of weight change across the life course. Emerging prospective cohort studies are now focusing on the effects of weight change over the life course on cardiometabolic risk [4–9]. It has been shown that weight change patterns in different life periods are important factors contributing to the variation observed in cardiometabolic risk [4–13]. Specifically, weight gain during earlier adulthood (i.e. between 25 and 40 years of age) has been associated with a higher risk of diabetes, cardiovascular disease (CVD), CVD mortality, and certain types of cancer than weight gain in later life (i.e. between 40 and 55 years of age) [4–7]. Weight change is common during an individual’s adulthood since adults are more likely to cumulate their excessive adiposity and thus accumulate weight rapidly from young adulthood to midlife, whereas weight begins to stabilize or even decrease across midlife to late adulthood [14]. The peak of obesity rates is trending increasingly towards younger ages [15], indicating that later-born generations accumulate greater exposure to obesity throughout their lives. Therefore, it is crucial to track how weight has changed from young adulthood through midlife to late adulthood over time. This important topic is not well studied but has clinical and public health ramifications.

The National Health and Nutrition Examination Surveys (NHANES) have routinely asked questions about participants’ weight histories (weight at age 25 [young adulthood] and weight at 10 years before their age at survey time [midlife]), and measured weight at the time of survey (late adulthood). Therefore, data from these surveys can be used to examine trends in weight change patterns across life course and their sex and racial/ethnic disparities among US adults.

## Methods

### Study design and population

Data for this study were drawn from the NHANES, which used a complex stratified, multistage probability cluster sampling design to ensure that the sample is nationally representative of the civilian, noninstitutionalized US population. Full details of the survey have been described elsewhere [16]. Briefly, NHANES was conducted periodically before 1999 and on a continuous basis thereafter in 2-year cycles. Participants were interviewed at home for basic sociodemographic and health-related information. After the in-home interview, participants are invited to attend a mobile examination center, where they underwent a set of standardized physical examinations and laboratory measurements. The survey procedures were reviewed and approved by the National Center for Health Statistics ethics review board (this is a full name of ethics committee) in accordance with the ethical standards laid down in the 1964 Declaration of Helsinki and its later amendments. Written informed consent was obtained from all participants.

In the present study, we used data from the NHANES III (1988–1994) and nine 2-year NHANES cycles (between 2001 and 2002 and 2017 to 2018). The examination response rate for adults during these cycles ranges from 77.6% in NHANES III to 47.7% in 2017–2018. The Centers for Disease Control and Prevention has evaluated the data and conducted enhanced weighting adjustment for the decreased response rate in recent years to minimize potential nonresponse bias.

We combined the nine 2-year NHANES cycles into three 6-year periods (2001–2006, 2007–2012, and 2013–2018) to produce estimates with greater statistical reliability and smaller sampling error. We included participants aged 35 or over at the time of survey. We excluded pregnant women and individuals with missing observations for BMI at the time of survey. We also excluded individuals missing self-reported weight at age 25 or 10 years before the survey time. Ultimately, a sample size of 48,969 participants (1988–1994 [n = 11,773], 2001–2006 [n = 11,077], 2007–2012 [n = 13,290], and 2013–2018 [n = 12,829]) remained for analysis.

### Assessments of weight and weight history

Respondents were asked to recall their weight at age 25 and 10 years before the survey time during the home interview. Weight and height at the time of survey were measured by trained staff following standardized protocols. Body mass index (BMI) at age 25 years (BMI_age25_, early adulthood), at 10 years before the survey time (BMI_10prior_, midlife), and at the time of survey (BMI_survey_, late adulthood) were calculated as weight in kilograms divided by height in meters squared. To account for the possibility of height decline with age, self-reported height at age 25 was used to calculate BMI_age25_ for participants who were 50 years or older at the time of survey. In other cases, measured height was used to calculate BMI. BMI values at each time were categorized into normal weight (18.5–24.9), overweight (25.0-29.9), and obesity (≥ 30.0) according to clinical guidelines [17].

### Outcomes

The primary outcomes were BMI change patterns. BMI change patterns were generated to capture weight change from young adulthood through midlife to late adulthood of an individual. Specifically, BMI changes between BMI_age25_ and BMI_10prior_ captured weight change from early adulthood to midlife; BMI changes between BMI_10prior_ and BMI_survey_ captured weight change from midlife to late adulthood; BMI changes between BMI_age25_ and BMI_survey_ captured weight change across the whole adulthood.

For each life stage, we defined four BMI change groups based on BMI (kg/m^2^) at two time points (eTable 1) following a recent study by Stokes et al. [6]: stable non-obesity (BMI < 30.0 at both times), weight loss (BMI ≥ 30.0 at younger age and < 30.0 later), weight gain (BMI < 30.0 at younger age and ≥ 30.0 later), and stable obesity (BMI ≥ 30.0 at both times).

Reports suggested that rates of weight change vary with age and can accelerate the development of diabetes and CVD [18]. We also examined rates of weight change. For each time interval, we calculated the rate of weight change by dividing the weight change (defined as weight at later age minus weight at younger age) by the time difference in years between the time intervals.

### Other variables

For all surveys, all participants were asked to complete a standardized questionnaire which collected information on age, sex, race/ethnicity, smoking habits, and histories of current and previous illness.

Self-reported race/ethnicity was categorized as Mexican American, non-Hispanic Black, non-Hispanic White, and other. Self-reported smoking status was categorized as never-smokers, former smokers, and current smokers. Never smokers were defined as smoke less than 100 cigarettes in their lifetime.

### Data analysis

Complex survey procedures in SAS 9.4 (SAS Institute Inc., Cary, North Carolina) were performed for all analyses. According to the NHANES analytic guidelines, sample weights, which adjusted for the unequal selection probabilities due to the sample design, nonresponse, and noncoverage were incorporated to produce nationally representative estimates. Both NHANES III and the continuous NHANES target the civilian noninstitutionalized U.S. population. Like other reports [19–21], weighting was conducted separately within each survey period (NHANES III and NHANES continuous) to make population in each survey period to be nationally representative. To maximize the comparability between surveys, means, and percentages for participants aged 35 years or older were age-adjusted by the direct method to the 2000 US Census population data using the age categories of 35–49 years, 50–64 years, and ≥ 65 years. In 2000, the proportion of adults aged 35–49 years, 50–64 years, and ≥ 65 years or older were 0.459, 0.294, and 0.247, respectively. Continuous variables were expressed as means (95% confidence intervals [CI]). Categorical variables were expressed as percentages (95% CI). Standard errors of the means and percentages used to calculate 95% CI were estimated by Taylor Series Linearization. For sensitivity analysis, we also calculated the unadjusted values and trends. We assessed linear trends using survey-weighted logistic (weight change patterns), or linear (rates of weight change) regression models with survey as a continuous independent variable. Age, sex, and race/ethnicity were adjusted in these models except when used as a stratified variable. To assess statistical heterogeneity of trends by subgroups, a survey-weighted Wald test was used to test for an interaction term between survey and categorical variables.

For sensitivity analyses, we repeated our trend analyses when participants aged 45 or older at the time of survey were included to avoid the issue that middle and late adulthood being either the same or very close together for some participants. Furthermore, we investigated trends in weight change patterns from 1988 to 1994 to 2013–2018 among participants aged between 45 and 65 years at the time of survey and among participants aged ≥ 65 years at the time of survey, since there are known differences in weight change patterns with older age.

Two-sided *P* values less than 0.05 were considered statistically significant.

## Results

### Participant characteristics

Table 1 presented the characteristics of the sample from each survey. From 1988 to 2018, the proportion of younger adults and respondents from non-Hispanic White individuals declined, while the proportion of other races/ethnicities increased notably; The estimated prevalence of obesity at age 25, at 10 years before the survey time, and at the time of survey increased significantly (*P* < 0.001 for all trends). BMI_age25,_ BMI_10prior,_ and BMI_survey_ increased from 1988 to 1994 to 2013–2018 (*P* < 0.001 for all trends). Differences between BMI_age25_ and BMI_10prior_ or between BMI_age25_ and BMI_survey_ presented in earlier surveys were larger than that in later surveys.

**Table 1:** Characteristics of participants by National Health and Nutrition Examination Survey, 1988–2018

	1988–1994 n = 11,773	2001–2006 n = 11,077	2007–2012 n = 13,290	2013–2018 n = 12,829	*P* value for trend
Age, y, mean (95% CI)	53.8 (53.7–54.0)	54.1 (53.9–54.2)	54.1 (53.9–54.2)	54.0 (53.9–54.2)	< 0.001
Age group, y, % (95% CI)					
35–49	46.4 (43.9–48.9)	45.1 (42.8–47.4)	40.1 (38.4–42.0)	35.8 (34.1–37.5)	< 0.001
50–64	27.9 (26.3–29.4)	31.6 (30.1–33.2)	35.9 (34.5–37.2)	36.8 (35.4–38.2)	< 0.001
≥ 65	25.7 (23.5–28.0)	23.3 (21.7–25.0)	24.0 (22.9–25.1)	27.4 (25.8–29.1)	0.185
Sex, % (95% CI)					
Men	46.4 (45.1–47.7)	47.6 (46.8–48.3)	47.5 (46.5–48.5)	47.2 (46.3–48.2)	0.371
Women	53.6 (52.3–54.9)	52.4 (51.7–53.2)	52.5 (51.5–53.5)	52.8 (51.8–53.7)	0.371
Race/ethnicity, % (95% CI)					
Mexican American	3.8 (3.2–4.4)	5.5 (4.2–6.9)	7.0 (5.1–8.9)	8.4 (6.3–10.4)	< 0.001
Non-Hispanic Black	9.9 (8.8–11.0)	10.5 (8.4–12.5)	10.7 (8.7–12.8)	11.0 (8.9–13.0)	0.465
Non-Hispanic White	79.1 (76.9–81.4)	75.9 (72.6–79.2)	70.8 (66.9–74.7)	65.8 (61.9–69.7)	< 0.001
Other	7.1 (5.5–8.8)	8.1 (6.4–9.8)	11.5 (9.6–13.4)	14.9 (13.1–16.7)	< 0.001
Weight, kg, mean (95% CI)					
At age 25	65.4 (64.9–65.9)	67.9 (67.3–68.4)	68.7 (68.3–69.1)	70.1 (69.6–70.6)	< 0.001
At 10 years before survey	72.2 (71.6–72.7)	75.9 (75.3–76.5)	77.5 (76.9–78.0)	79.8 (79.0-80.5)	< 0.001
At the time of survey	76.7 (76.1–77.4)	81.7 (81.0-82.4)	82.5 (81.9–83.1)	84.2 (83.5–85.0)	< 0.001
Body mass index, kg/m^2^, mean (95% CI)					
At age 25	23.1 (22.9–23.2)	23.3 (23.2–23.5)	23.6 (23.5–23.8)	24.2 (24.1–24.4)	< 0.001
At 10 years before survey	25.5 (25.3–25.7)	26.6 (26.4–26.7)	27.2 (27.0-27.3)	28.2 (27.9–28.4)	< 0.001
At the time of survey	27.2 (26.9–27.4)	28.7 (28.4–28.9)	29.0 (28.8–29.2)	29.8 (29.6–30.1)	< 0.001
Obesity, % (95% CI)					
At age 25	5.1 (4.2-6.0)	6.5 (5.8–7.2)	7.3 (6.6-8.0)	10.2 (9.1–11.2)	< 0.001
At 10 years before survey	14.7 (13.5–16.0)	20.3 (19.0-21.7)	24.5 (23.1–26.0)	30.4 (28.9–32.0)	< 0.001
At the time of survey	25.3 (23.4–27.1)	34.2 (32.6–35.7)	36.5 (35.1–37.9)	42.0 (40.1–43.8)	< 0.001
Non-smokers, % (95% CI)	42.7 (40.9–44.5)	48.0 (46.3–49.7)	53.0 (51.6–54.5)	54.7 (53.1–56.4)	< 0.001

The means and percentages were weighted

### Trends in weight change patterns across life course

**Fig. 1:**
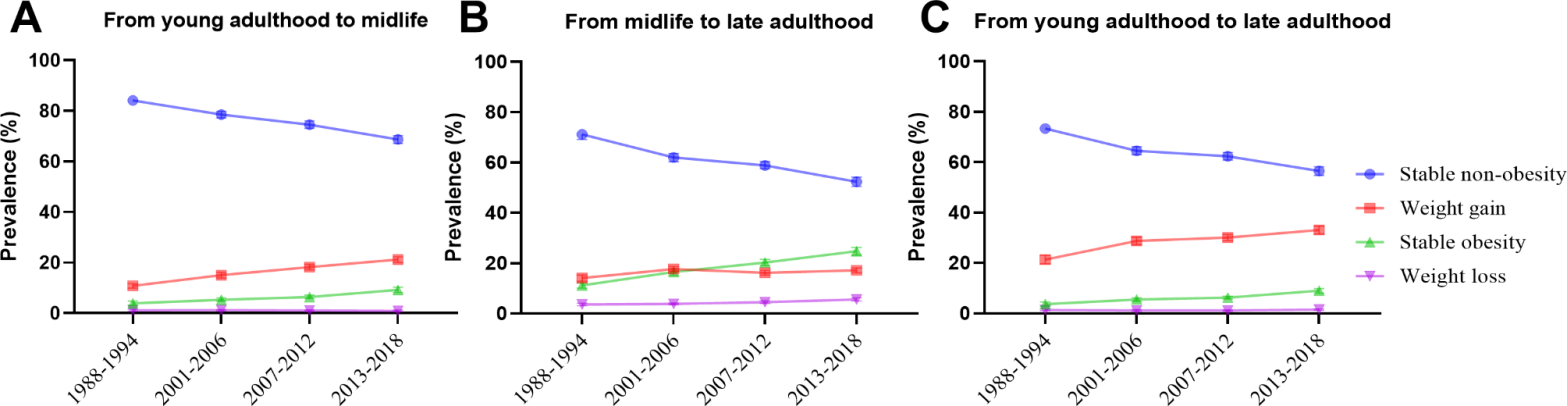
Trends in weight change patterns across life course in all participants, 1988–2018. The percentages were survey-weighted

Trends in weight change patterns were shown in Fig. 1 (overall) and eTable 2 (by race/ethnicity). The age-adjusted prevalence of stable non-obesity between young adulthood and midlife declined from 84.1% (82.9-85.3%) in 1988–1994 to 68.7% (67.1-70.2%) in 2013–2018 (*P* < 0.001 for trend), and between midlife and late adulthood from 71.2% (69.2-73.1%) to 52.4% (50.5-54.2%) (*P* < 0.001 for trend) (Fig. 1). The age-adjusted prevalence of weight gain between young adulthood and midlife increased from 10.8% (9.9-11.6%) in 1988–1994 to 21.2% (20-22.3%) in 2013–2018 (*P* < 0.001 for trend), and between midlife and late adulthood from 14.1% (12.9-15.3%) to 17.2% (6.2-18.1%) (*P* = 0.002 for trend). Of note, the magnitude of increase in the percentage of weight gain from young adulthood to midlife (10.4-percentage point increase) was much greater than that from midlife to late adulthood (3.1-percentage point increase) (*P* for interaction < 0.001). The age-adjusted prevalence of stable obesity between young adulthood and midlife increased from 3.9% (3.1-4.8%) in 1988–1994 to 9.2% (8.2-10.3%) in 2013–2018 (*P* < 0.001 for trend), and between midlife and late adulthood from 11.2% (10.1-12.2%) to 24.8% (23.3-26.3%) (*P* < 0.001 for trend). The increase in the prevalence of stable obesity from midlife to late adulthood (13.7-percentage point increase) was greater than that from young adulthood to midlife (5.3-percentage point increase) (*P* for interaction < 0.001) (Fig. 1). The prevalence of stable non-obesity declined and the prevalence of stable obesity increased over time from midlife to late adulthood in all race/ethnicity subgroups (*P* < 0.01 for trend). (eTable 2). The prevalence of weight gain increased over time from midlife to late adulthood in all race/ethnicity subgroups except for Mexican American individuals (eTable 2).

The age-adjusted prevalence of weight loss from young adulthood to midlife did not change significantly, while it slightly increased from midlife to late adulthood (3.6% [3.1-4.1%] in 1988–1994 versus 5.6% [5.0-6.3%] in 2013–2018; *P* < 0.001 for linear trend).

Trends in weight change patterns across the whole adulthood were extremely similar to those from young adulthood to midlife (Fig. 1 and eTable 2).

Since accumulating evidence has shown sex disparities in obesity as well as the epidemiology, progression, and outcomes of chronic disease such as diabetes and cardiovascular disease [22, 23], we stratified the data by sex (Table 2). The prevalence of stable non-obesity declined and the prevalence of weight gain as well as stable obesity from young adulthood through midlife to late adulthood increased over time for both men and women. Of note, the magnitude of decrease in the percentage of stable non-obesity, and the magnitude of increase in the percentage of weight gain from young adulthood through midlife to late adulthood was greater in men than in women (all *P* for interaction < 0.01). The age-adjusted prevalence of weight loss from young adulthood to midlife did not change in men (from 1.7 [1.1-2.2%] in 1988–1994 to 0.9% [0.5-1.2%] in 2013–2018; *P* = 0.003 for trend), while it slightly increased in women (*P* = 0.03 for trend).

**Table 2:** Trends in weight change patterns across life course in men and women, 1988–2018

	Survey-weighted percentage (95% CI)				
	1988–1994	2001–2006	2007–2012	2013–2018	*P* value for trend
Men^a^					
From young adulthood to midlife					
Stable non-obesity	82.4 (80.8–84.0)	77.2 (75.2–79.2)	72.1 (70.3–73.9)	66.1 (64.3–67.9)	< 0.001
Weight gain	11.4 (10.2–12.6)	15.5 (13.9–17.1)	19.8 (18.4–21.1)	22.6 (21.1–24.1)	< 0.001
Stable obesity	4.6 (3.4–5.7)	5.8 (4.8–6.8)	7.1 (6.1–8.1)	10.4 (9.0-11.8)	< 0.001
Weight loss	1.7 (1.1–2.2)	1.4 (1.0-1.9)	1.0 (0.7–1.4)	0.9 (0.5–1.2)	0.003
From midlife to late adulthood					
Stable non-obesity	72.3 (70.2–74.4)	63.0 (61.0–65.0)	58.5 (56.7–60.3)	52.3 (49.9–54.6)	< 0.001
Weight gain	11.9 (10.5–13.3)	15.8 (14.5–17.0)	14.5 (13.3–15.7)	14.7 (13.2–16.3)	0.034
Stable obesity	11.0 (9.8–12.3)	16.9 (15.0-18.9)	21.5 (19.8–23.2)	26.3 (24.3–28.2)	< 0.001
Weight loss	4.7 (3.8–5.6)	4.3 (3.6-5.0)	5.4 (4.6–6.3)	6.7 (5.7–7.8)	0.002
From young adulthood to late adulthood					
Stable non-obesity	75.2 (73.4–77.0)	65.6 (63.6–67.7)	62.6 (60.6–64.6)	57.1 (54.8–59.4)	< 0.001
Weight gain	18.6 (16.8–20.5)	26.7 (24.8–28.6)	29.1 (27.5–30.7)	31.3 (29.3–33.2)	< 0.001
Stable obesity	4.0 (2.9–5.1)	6.1 (5.1–7.1)	6.8 (5.8–7.8)	9.8 (8.4–11.2)	< 0.001
Weight loss	2.2 (1.5–2.8)	1.5 (1.0–2.0)	1.5 (1.1–1.9)	1.8 (1.3–2.3)	0.322
Women ^a^					
From young adulthood to midlife					
Stable non-obesity	85.7 (84.1–87.2)	79.7 (78.3–81.0)	76.7 (75.0-78.5)	71.0 (68.7–73.3)	< 0.001
Weight gain	10.3 (9.1–11.5)	14.5 (13.4–15.7)	16.7 (15.2–18.2)	19.9 (18.1–21.7)	< 0.001
Stable obesity	3.4 (2.5–4.2)	4.9 (4.2–5.7)	5.7 (4.8–6.5)	8.2 (7.1–9.3)	< 0.001
Weight loss	0.7 (0.3–1.1)	0.9 (0.5–1.2)	0.9 (0.5–1.3)	1.0 (0.5–1.4)	0.386
From midlife to late adulthood					
Stable non-obesity	70.1 (67.6–72.5)	61.0 (59.1–62.9)	59.3 (57.3–61.3)	52.5 (50.0–55.0)	< 0.001
Weight gain	16.1 (14.5–17.7)	19.5 (18.2–20.9)	17.8 (16.5–19.0)	19.3 (18.1–20.6)	0.009
Stable obesity	11.3 (9.8–12.7)	16.0 (14.7–17.3)	19.2 (17.6–20.7)	23.5 (21.5–25.5)	< 0.001
Weight loss	2.6 (2.0-3.1)	3.5 (3.0–4.0)	3.7 (3.2–4.3)	4.7 (3.8–5.5)	0.002
From young adulthood to late adulthood					
Stable non-obesity	71.9 (69.5–74.2)	63.5 (61.6–65.4)	62.3 (60.4–64.2)	55.9 (53.7–58.1)	< 0.001
Weight gain	23.9 (21.8–26.0)	30.7 (29.0-32.3)	31.0 (29.3–32.7)	34.7 (32.9–36.5)	< 0.001
Stable obesity	3.4 (2.6–4.3)	4.9 (4.1–5.8)	5.9 (5.0-6.7)	8.2 (7.1–9.3)	< 0.001
Weight loss	0.8 (0.5–1.1)	0.9 (0.6–1.2)	0.9 (0.6–1.1)	1.2 (0.8–1.6)	0.267

^a^*P* value for trend was adjusted for age, and race/ethnicity

The unadjusted prevalence and trends in weight change patterns were shown in eTable 3–4.

The characteristics of participants presenting with weight loss from young adulthood to midlife were shown in eTable 5. From 1988 to 2018, the proportion of men showing weight loss declined. BMI_age25,_ BMI_10prior,_ and BMI_survey_ for participants showing weight loss increased from 1988 to 1994 to 2013–2018 (*P* < 0 0.05 for all trends).

### Trends in the rates of weight change across life course

The rate of weight gain from young adulthood to midlife increased from 0.53 kg/year (0.48–0.58 kg/year) in 1988–1994 to 0.69 kg/year (0.64–0.74 kg/year) in 2013–2018 (*P* < 0.001 for trend) (Table 3). Conversely, the rate of weight gain increased slower from midlife to late adulthood (from 0.14 kg/year [0.12–0.15 kg/year] to 0.13 kg/year [0.12–0.14 kg/year]; *P* = 0.061). The rate of weight gain across the whole adulthood increased from 0.51 kg/year (0.48–0.53 kg/year) in 1988–1994 to 0.59 kg/year (0.57–0.62 kg/year) in 2013–2018 (*P* < 0.001 for trend). The faster rate of weight gain from young adulthood to midlife than that from midlife to late adulthood was also noted in women, Mexican Americans, and non-Hispanic Whites.

**Table 3:** Trends in the rates of weight gain (kg/year) across life course, 1988–2018

	Survey-Weighted mean (95% CI)				
	1988–1994	2001–2006	2007–2012	2013–2018	*P* value for trend
All^a^					
From young adulthood to midlife	0.53 (0.48–0.58)	0.54 (0.50–0.58)	0.60 (0.56–0.64)	0.69 (0.64–0.74)	< 0.001
From midlife to late adulthood	0.14 (0.12–0.15)	0.16 (0.15–0.17)	0.14 (0.13–0.15)	0.13 (0.12–0.14)	0.061
From young adulthood to late adulthood	0.51 (0.48–0.53)	0.59 (0.56–0.61)	0.58 (0.56–0.60)	0.59 (0.57–0.62)	< 0.001
Men^b^					
From young adulthood to midlife	0.58 (0.50–0.66)	0.52 (0.48–0.56)	0.59 (0.53–0.64)	0.66 (0.60–0.73)	0.08
From midlife to late adulthood	0.09 (0.07–0.10)	0.12 (0.11–0.13)	0.11 (0.10–0.12)	0.10 (0.08–0.11)	0.668
From young adulthood to late adulthood	0.42 (0.37–0.46)	0.50 (0.47–0.53)	0.51 (0.49–0.53)	0.52 (0.49–0.55)	0.001
Women^b^					
From young adulthood to midlife	0.48 (0.40–0.57)	0.57 (0.51–0.63)	0.62 (0.56–0.67)	0.71 (0.63–0.79)	0.001
From midlife to late adulthood	0.18 (0.17–0.20)	0.20 (0.19–0.21)	0.17 (0.16–0.18)	0.16 (0.14–0.18)	0.005
From young adulthood to late adulthood	0.59 (0.54–0.63)	0.67 (0.64–0.70)	0.64 (0.61–0.66)	0.66 (0.62–0.70)	0.03
Mexican American^c^					
From young adulthood to midlife	0.62 (0.53–0.71)	0.58 (0.48–0.68)	0.70 (0.59–0.82)	0.82 (0.71–0.94)	0.003
From midlife to late adulthood	0.15 (0.13–0.16)	0.17 (0.15–0.19)	0.15 (0.12–0.18)	0.13 (0.11–0.16)	0.137
From young adulthood to late adulthood	0.60 (0.57–0.64)	0.62 (0.57–0.66)	0.62 (0.57–0.67)	0.65 (0.61–0.68)	0.171
Non-Hispanic Black^c^					
From young adulthood to midlife	0.67 (0.59–0.76)	0.66 (0.57–0.75)	0.80 (0.71–0.89)	0.75 (0.65–0.84)	0.109
From midlife to late adulthood	0.17 (0.16–0.19)	0.22 (0.20–0.24)	0.20 (0.18–0.22)	0.20 (0.18–0.22)	0.305
From young adulthood to late adulthood	0.66 (0.62–0.69)	0.77 (0.73–0.81)	0.76 (0.71–0.80)	0.74 (0.70–0.79)	0.008
Non-Hispanic White^c^					
From young adulthood to midlife	0.51 (0.44–0.58)	0.54 (0.48–0.59)	0.59 (0.52–0.65)	0.66 (0.58–0.74)	0.001
From midlife to late adulthood	0.13 (0.11–0.14)	0.15 (0.14–0.16)	0.14 (0.12–0.15)	0.12 (0.10–0.14)	0.2
From young adulthood to late adulthood	0.48 (0.44–0.51)	0.56 (0.53–0.59)	0.56 (0.53–0.58)	0.57 (0.53–0.60)	< 0.001
Other races/ethnicities ^c^					
From young adulthood to midlife	0.59 (0.35–0.82)	0.48 (0.35–0.61)	0.58 (0.48–0.68)	0.70 (0.62–0.77)	0.293
From midlife to late adulthood	0.16 (0.11–0.20)	0.15 (0.12–0.18)	0.13 (0.11–0.14)	0.12 (0.10–0.14)	0.07
From young adulthood to late adulthood	0.55 (0.47–0.63)	0.54 (0.49–0.58)	0.53 (0.49–0.57)	0.57 (0.52–0.62)	0.584

^a^*P* value for trend was adjusted for age, sex, and race/ethnicity;

^b^*P* value for trend was adjusted for age and race/ethnicity;

^c^*P* value for trend was adjusted for age and sex

The unadjusted means and trends in the rates of weight change were shown in eTable 6.

The rate of weight gain or loss since age 25 could potentially be different despite similar absolute weight changes. We repeated our trend analyses stratified by age at the time of the survey. Results were essentially the same in each age group (eTable 7). Weight gain patterns for those over 65 years were substantially different from the younger age groups. For example, values of the rate of weight gain in the younger age groups are generally greater than 0.60 kg/year, while in older adults, values are generally less than 0.30 kg/year.

### Sensitivity analyses

We repeated our trend analyses when limiting our sample size to participants aged 45 or older at the time of survey, results were essentially the same (eTable 8–9). Moreover, to avoid the effect of older age on weight change, we investigated trends in weight change patterns among participants aged between 45 and 65 years at the time of survey, results were also remarkably the same (eTable 10–11). We also repeated our trend analyses when limiting our sample size to participants aged ≥ 65 years at the time of survey, results were essentially the same (eTable 12).

## Discussion

This study used the NHANES survey data to examine trends in weight change patterns and their sex or racial/ethnic disparities among US adults from 1988 to 1994 to 2013–2018. We found that from young adulthood through midlife to late adulthood, the age-adjusted prevalence of stable non-obesity decreased, whereas the prevalence of weight gain, as well as stable obesity increased over time, with little variation among sex or racial/ethnic groups. To our knowledge, our novel findings represent the most comprehensive evaluation of trends in how the process of obesity development across adulthood has changed among US adults.

Our findings that the prevalence of weight gain from young adulthood through middle to late adulthood increased significantly from 1988 to 1994 to 2013–2018, with greater increases from young adulthood to midlife than from midlife to late adulthood. Our study also showed that the prevalence of stable obesity from young adulthood through middle adulthood to late adulthood increased significantly from 1988 to 1994 to 2013–2018, with smaller increases from young adulthood to midlife than from midlife to late adulthood. These findings are in agreement with ①other secular trend studies showing an increasing BMI in younger individuals over time [15, 24], and ②other studies showing that people in young adulthood having obesity are likely to have obesity or gain more weight in later life [12, 25–27]. Our results suggest that the number of individuals with obesity in young adulthood was much greater than the number of young people born in earlier years and, if the observed trends continue, the majority of people are likely to develop obesity at younger ages and thus younger individuals born more recently are accumulating greater exposure to obesity throughout their lives. Studies showed that young adulthood represents a time period in which obesity, and its cumulative exposure lay an important foundation for a wide range of cardiometabolic disease in later life [12, 28, 29]. Therefore, it is obviously important to develop policies and programs aimed at preventing early obesity onset, and thus reducing lifetime obesity exposure to reduce future cardiometabolic disease burden.

In the present study, our results that weight gain patterns in older adults were substantially different from that in younger adults and that declining trends in the age-adjusted prevalence of stable non-obesity from young adulthood through middle adulthood to late adulthood from 1988 to 1994 to 2013–2018 might be due to the faster rates of weight gain from young adulthood to midlife, which is likely linked to an earlier development of obesity and thus induce an increasing number of young people suffering from weight gain.

The low prevalence of weight loss which remained essentially unchanged, and the declining trend in the prevalence of stable non-obesity, and statistically increasing trend in the prevalence of weight gain from young adulthood to midlife over time as well as the dramatic increasing trend in the prevalence of stable obesity from midlife to late adulthood observed in our study may explain the continuously high obesity prevalence (more than one-third of the population) [1, 3] and increasing trends in obesity over time [3].

In the present study, we identified significant sex differences in weight change patterns: ①Men had a greater magnitude of decrease in the prevalence of stable non-obesity, and greater magnitude of increase in the percentage of weight gain from young adulthood through midlife to late adulthood than women; ②less number of men showing weight loss in recent surveys. There is a well-recognized diabetes and cardiovascular disease risk disparity by sex, with lower risk in women than in men [22, 23]. Our findings may indicate that men tended to put on more weight in their early life and thus inducing a worse cardiovascular risk profiles and greater diabetes and cardiovascular incidence.

In the present study, we also noted racial/ethnicity differences in weight change patterns. Evidence has shown racial/ethnicity disparities in the epidemiology, progression, and outcomes of diabetes and cardiovascular disease [30, 31]. Our findings may offer new insights into the risk disparity in diabetes and cardiovascular disease by race/ethnicity.

The prevalence of weight gain and stable obesity from young adulthood through midlife to late adulthood increased significantly in almost all subgroups, suggesting an obesogenic environment and broad behavioral, sociocultural, and economic causes [32–37]. For example, environmental endocrine disruptors, changes in food environment and food systems that increase availability, accessibility, and affordability of energy-dense foods, increasingly mechanized transportation and labor that promote inactivity, insufficient sleep, and occupational status (stress, and shift work) have been considered as major drivers [32–37].

The main strength of this study is the use of data from NHANES, which collected weight information from young adulthood through midlife to late adulthood, and thus facilitating evaluation of the dynamic aspects of weight progression across age and time. We were able to comprehensively evaluate trends in how the process of weight change from young adulthood through midlife to late adulthood has happened. By the inclusion of more recently survey, the present study further demonstrates how the later-born individuals have changed their weight (compared to earlier-born individuals) because of earlier exposure to the obesogenic environment. Since weight gain from young adulthood to midlife reflects mainly fat mass accumulation, while weight loss from midlife to late adulthood usually reflects a decrease in lean mass [38], assessment of weight change patterns in different life periods can reflect individual differences in lean mass, fat, as well as other aspects of body composition including bone mass, muscle loss, etc. In addition, a large sample size allows us to examine the potential differences by sociodemographic subgroups.

This study has several limitations. Firstly, we relied on historic, self-reported weight measures, which may introduce classification error. Several validation studies evidenced a relatively high level of agreement between self-reported weight and measured weight [39]. The recalled weight in early life can be used as a valid measure in life course epidemiological analysis [40]. However, several reports noted that self-reporting tends to overestimate height and underestimate weight, and thus BMI and the obesity prevalence will be underestimated [41, 42]. An underestimation of weight and an overestimation of height causes a multiplication of errors and, thus, may contribute to incorrect indication of body fat distribution as well as diagnostic decisions. Secondly, for adults aged 35 years and slightly older at the time of the survey, their middle adulthood and late adulthood weight would be either the same or very close together. However, we did a sensitivity analysis by including participants aged 45 or older at the time of survey, and the results were essentially the same. Thirdly, weight change patterns were assessed by BMI status and not by measurements of body fatness. Body fatness at a given BMI or weight may vary by sex and race/ethnicity [43–45]. However, evidence showed that BMI and body fatness are highly correlated [44].

## Conclusions

This study provides the trends in how weight has changed across the whole adulthood in the US. Our results demonstrate that from 1988 to 1994 to 2013–2018, the age-adjusted prevalence of stable non-obesity decreased, whereas the prevalence of weight gain as well as stable obesity increased across the whole adulthood, with little variation among sex or racial/ethnic groups. Therefore, more young people are encountering obesity and accumulate greater obesity exposure across their lives than young people born in earlier years. These findings may inform discussions on areas for greater attention and corresponding opportunities for improving weight management in the US.

### Electronic supplementary material

Below is the link to the electronic supplementary material.

Supplementary Material 1

## Data Availability

Data are available from the NHANES website (wwwn.cdc.gov/nchs/nhanes/default.aspx). The manuscript’s guarantor (Du) affirms that the manuscript is an honest, accurate, and transparent account of the study being reported; that no important aspects of the study have been omitted; and that any discrepancies from the study as planned (and, if relevant, registered) have been explained.

## List of abbreviations

CVD: cardiovascular disease
NHANES: the National Health and Nutrition Examination Surveys
BMI: body mass index
