# Supplementary material for: Trends in weight change patterns across life course among US adults, 1988–2018: population-based study
Source: BMC Public Health. 2023 Nov 6;23:2168. doi: 10.1186/s12889-023-17137-x (PMC10626664; doi:10.1186/s12889-023-17137-x)
Supplement: Supplementary file 1 — Supplementary Material 1 [file 12889_2023_17137_MOESM1_ESM.docx]

**Supplement Materials**

**Supplementary tables**

eTable 1 The definition of weight change patterns using the life stage between age 25 years and 10 years before the survey time as an example

eTable 2 Trends in weight change patterns across life course by race/ethnicity, 1988-2018 (adjusted)

eTable 3 Trends in weight change patterns across life course, 1988-2018 (unadjusted)

eTable 4 Trends in weight change patterns across life course by sex and race/ethnicity, 1988-2018 (unadjusted)

eTable 5 Characteristics of participants presenting with weight loss, 1988-2018 (adjusted)

eTable 6 Trends in the rates of weight gain (kg/year) across life course, 1988-2018 (unadjusted)

eTable 7 Trends in the rates of weight gain (kg/year) across life course stratified by age at the time of the survey, 1988-2018 (adjusted)

eTable 8 Trends in weight change patterns across life course among participants aged 45 or older at the time of survey, 1988-2018 (adjusted)

eTable 9 Trends in the rates of weight gain (kg/year) across life course among participants aged 45 or older at the time of survey, 1988-2018 (adjusted)

eTable 10 Trends in weight change patterns across life course among participants aged between 45 and 65 years at the time of survey, 1988-2018 (adjusted)

eTable 11 Trends in the rates of weight gain (kg/year) across life course among participants aged between 45 and 65 years at the time of survey, 1988-2018 (adjusted)

eTable 12 Trends in weight change patterns across life course among participants aged≥ 65 years at the time of survey, 1988-2018 (adjusted)

**eTable 1 The definition of weight change patterns using the life stage between age 25 years and 10 years before the survey time as an example**

| **Body mass index at age 25 years** | **Body mass index at 10 years before survey time** | |
| --- | --- | --- |
|  | 18.5-29.9 kg/m^2^ | ≥30 kg/m^2^ |
| 18.5-29.9 kg/m^2^ | Stable non-obesity | Weight gain |
| ≥30 kg/m^2^ | Weight loss | Stable obesity |

eTable 2 Trends in weight change patterns across life course by race/ethnicity, 1988-2018

|  |  | Survey-weighted percentage (95% CI) | | | | |
| --- | --- | --- | --- | --- | --- | --- |
|  |  | 1988-1994 | 2001-2006 | 2007-2012 | 2013-2018 | *P* value for trend |
| Mexican American ^a^ | | | | | | |
| From young adulthood to midlife | | | | | | |
|  | Stable non-obesity | 78.4 (76.1-80.8) | 77.6 (75.3-79.9) | 68.3 (66.3-70.4) | 60.9 (57.7-63.9) | <0.001 |
|  | Weight gain | 16.37 (14.2-18.5) | 15.5 (13.6-17.3) | 23.2 (20.7-25.7) | 26.9 (23.1-30.2) | <0.001 |
|  | Stable obesity | 4.2 (3.4-5.1) | 5.4 (3.9-6.8) | 6.8 (4.9-8.6) | 10.9 (8.7-13.2) | <0.001 |
|  | Weight loss | 1.0 (0.5-1.4) | 1.6 (0.8-2.5) | 1.7 (0.9-2.4) | 1.3 (0.6-1.9) | 0.628 |
| From midlife to late adulthood | | | | | | |
|  | Stable non-obesity | 61.3 (58.9-63.8) | 58.9 (56.2-61.5) | 49.2 (45.8-52.5) | 41.8 (38.8-44.8) | <0.001 |
|  | Weight gain | 17.5 (15.9-19.1) | 20.2 (17.9-22.6) | 20.9 (17.7-24.1) | 19.7 (17.4-21.9) | 0.188 |
|  | Stable obesity | 14.7 (12.7-16.8) | 15.8 (13.7-17.9) | 23.3 (21.4-25.2) | 30.4 (27.3-33.5) | <0.001 |
|  | Weight loss | 6.4 (5.5-7.4) | 5.1 (4.2-6.0) | 6.6 (5.2-8.0) | 8.1 (6.5-9.7) | 0.007 |
| From young adulthood to late adulthood | | | | | | |
|  | Stable non-obesity | 66.4 (63.7-69.1) | 61.6 (58.8-64.5) | 54.1 (50.5-57.6) | 48.7 (45.9-51.3) | <0.001 |
|  | Weight gain | 28.5 (25.9-31.2) | 31.3 (28.7-33.9) | 37.6 (34.7-40.5) | 38.9 (35.4-42.4) | <0.001 |
|  | Stable obesity | 3.6 (2.7-4.5) | 4.9 (3.6-6.2) | 6.3 (4.4-8.2) | 10.9 (8.6-13.4) | <0.001 |
|  | Weight loss | 1.5 (1.0-2.1) | 2.1 (1.4-2.8) | 2.4 (1.4-2.7) | 1.5 (0.9-2.2) | 0.771 |
| Non-Hispanic Black ^a^ | | | | | | |
| From young adulthood to midlife | | | | | | |
|  | Stable non-obesity | 77.8 (75.5-80.1) | 72.5 (69.8-75.1) | 65.4 (62.6-68.2) | 64.4 (62.4-66.4) | <0.001 |
|  | Weight gain | 15.9 (14.2-17.6) | 18.4 (16.6-20.1) | 22.2 (20.2-24.2) | 22.4 (20.6-24.1) | <0.001 |
|  | Stable obesity | 5.4 (4.1-6.6) | 7.9 (6.4-9.5) | 11.3 (9.5-13.1) | 11.9 (10.5-13.4) | <0.001 |
|  | Weight loss | 0.9 (0.6-1.3) | 1.2 (0.7-1.8) | 1.1 (0.6-1.6) | 1.3 (0.7-1.9) | 0.353 |
| From midlife to late adulthood | | | | | | |
|  | Stable non-obesity | 60.5 (57.8-63.2) | 51.0 (48.6-53.5) | 45.5 (42.9-48.1) | 42.8 (40.1-45.4) | <0.001 |
|  | Weight gain | 17.8 (16.0-19.5) | 22.8 (21.2-24.3) | 20.8 (19.2-22.5) | 23.0 (21.1-24.9) | 0.001 |
|  | Stable obesity | 15.5 (13.8-17.2) | 21.2 (19.3-23.1) | 28.2 (25.8-30.7) | 28.9 (26.9-30.9) | <0.001 |
|  | Weight loss | 6.3 (5.3-7.2) | 4.9 (4.0-5.9) | 5.5 (4.2-6.7) | 5.3 (4.1-6.4) | 0.231 |
| From young adulthood to late adulthood | | | | | | |
|  | Stable non-obesity | 65.0 (62.7-67.3) | 53.8 (51.4-56.3) | 49.2 (46.5-51.9) | 46.2 (43.3-49.1) | <0.001 |
|  | Weight gain | 28.4 (26.6-30.3) | 36.8 (34.9-38.7) | 38.2 (35.7-40.6) | 40.1 (37.7-42.6) | <0.001 |
|  | Stable obesity | 4.7 (3.6-5.9) | 7.8 (6.1-9.5) | 10.9 (8.8-12.9) | 12.3 (10.9-13.8) | <0.001 |
|  | Weight loss | 1.8 (1.4-2.3) | 1.6 (1.0-2.2) | 1.7 (1.1-2.3) | 1.4 (0.8-1.8) | 0.407 |
| Non-Hispanic White ^a^ | | | | | | |
| From young adulthood to midlife | | | | | | |
|  | Stable non-obesity | 84.4 (82.9-86.0) | 78.6 (77.1-80.1) | 74.9 (73.1-76.7) | 67.8 (65.8-69.8) | <0.001 |
|  | Weight gain | 10.2 (9.2-11.2) | 15.1 (13.7-16.5) | 18.1 (16.7-19.5) | 21.9 (20.5-23.5) | <0.001 |
|  | Stable obesity | 4.2 (3.1-5.3) | 5.2 (4.3-5.9) | 6.1 (5.2-6.9) | 9.3 (7.6-10.9) | <0.001 |
|  | Weight loss | 1.2 (0.8-1.6) | 1.2 (0.9-1.5) | 0.9 (0.6-1.1) | 0.9 (0.5-1.4) | 0.182 |
| From midlife to late adulthood | | | | | | |
|  | Stable non-obesity | 72.6 (70.3-74.9) | 62.2 (60.5-63.9) | 60.2 (58.1-62.3) | 52.5 (50.2-54.8) | <0.001 |
|  | Weight gain | 13.1 (11.8-14.3) | 17.6 (16.6-18.6) | 15.4 (13.9-16.7) | 16.2 (14.8-17.5) | 0.004 |
|  | Stable obesity | 10.9 (9.6-12.3) | 16.4 (14.9-17.9) | 20.2 (18.6-21.8) | 25.8 (23.9-27.7) | <0.001 |
|  | Weight loss | 3.4 (2.8-4.0) | 3.8 (3.3-4.2) | 4.3 (3.6-4.9) | 5.6 (4.7-6.4) | <0.001 |
| From young adulthood to late adulthood | | | | | | |
|  | Stable non-obesity | 74.7 (72.5-76.9) | 65.0 (63.3-66.7) | 63.8 (61.8-65.9) | 56.5 (54.3-58.8) | <0.001 |
|  | Weight gain | 19.8 (17.8-21.8) | 28.4 (26.7-30.1) | 29.0 (27.3-30.8) | 32.9 (31.0-34.9) | <0.001 |
|  | Stable obesity | 3.9 (2.9-5.0) | 5.5 (4.6-6.4) | 6.2 (5.5-6.9) | 8.9 (7.4-10.5) | <0.001 |
|  | Weight loss | 1.5 (1.0-1.9) | 1.1 (0.7-1.4) | 0.9 (0.6-1.3) | 1.6 (1.1-2.1) | 0.825 |

^a^*P* value for trend was adjusted for age and sex.

**eTable 3 Trends in weight change patterns across life course, 1988-2018**

|  | **Unweighted percentage (95% CI)** | | | | |
| --- | --- | --- | --- | --- | --- |
|  | **1988-1994** | **2001-2006** | **2007-2012** | **2013-2018** | ***P* value for trend** |
| From young adulthood to midlife | | | | | |
| Stable non-obesity | 84.08 (82.86-85.30) | 78.43 (77.06-79.79) | 74.29 (72.81-75.78) | 67.87 (66.35-69.40) | <0.001 |
| Weight gain | 10.85 (9.99-11.70) | 15.10 (13.94-16.26) | 18.70 (17.53-19.88) | 22.77 (21.57-23.98) | <0.001 |
| Stable obesity | 3.92 (3.07-4.76) | 5.33 (4.69-5.97) | 6.07 (5.41-6.72) | 8.49 (7.54-9.44) | <0.001 |
| Weight loss | 1.15 (0.83-1.48) | 1.14 (0.88-1.40) | 0.93 (0.74-1.13) | 0.86 (0.60-1.13) | 0.092 |
| From midlife to late adulthood | | | | | |
| Stable non-obesity | 71.17 (69.19-73.15) | 61.79 (60.21-63.36) | 58.67 (57.20-60.15) | 52.03 (50.22-53.83) | <0.001 |
| Weight gain | 13.99 (12.80-15.18) | 17.80 (16.83-18.77) | 16.23 (15.30-17.16) | 16.64 (15.77-17.51) | 0.007 |
| Stable obesity | 11.21 (10.15-12.26) | 16.57 (15.22-17.92) | 20.56 (19.28-21.83) | 25.36 (23.92-26.80) | <0.001 |
| Weight loss | 3.63 (3.13-4.13) | 3.84 (3.46-4.23) | 4.54 (4.00-5.09) | 5.97 (5.27-6.67) | <0.001 |
| From young adulthood to late adulthood | | | | | |
| Stable non-obesity | 73.53 (71.69-75.37) | 64.32 (62.74-65.91) | 62.16 (60.66-63.66) | 56.49 (54.79-58.19) | <0.001 |
| Weight gain | 21.31 (19.63-23.00) | 28.92 (27.49-30.36) | 30.61 (29.37-31.85) | 33.81 (32.27-35.34) | <0.001 |
| Stable obesity | 3.71 (2.89-4.54) | 5.57 (4.91-6.23) | 6.08 (5.48-6.67) | 8.32 (7.40-9.24) | <0.001 |
| Weight loss | 1.44 (1.12-1.76) | 1.19 (0.91-1.46) | 1.15 (0.93-1.37) | 1.39 (1.14-1.63) | 0.739 |

*P* value for trend was unadjusted.

**eTable 4 Trends in weight change patterns across life course by sex and race/ethnicity, 1988-2018**

|  | | **Unweighted percentage (95% CI)** | | | | |
| --- | --- | --- | --- | --- | --- | --- |
|  | | **1988-1994** | **2001-2006** | **2007-2012** | **2013-2018** | ***P* for trend** |
| Men | | | | | | |
| From young adulthood to midlife | | | | | | |
|  | Stable non-obesity | 82.46 (80.81-84.11) | 77.25 (75.26-79.25) | 71.94 (70.07-73.82) | 65.30 (63.55-67.05) | <0.001 |
|  | Weight gain | 11.31 (10.01-12.62) | 15.43 (13.81-17.06) | 20.14 (18.69-21.60) | 24.24 (22.73-25.74) | <0.001 |
|  | Stable obese | 4.57 (3.43-5.72) | 5.86 (4.92-6.81) | 6.88 (5.87-7.88) | 9.67 (8.38-10.97) | <0.001 |
|  | Weight loss | 1.65 (1.12-2.19) | 1.45 (0.97-1.93) | 1.04 (0.70-1.37) | 0.79 (0.51-1.06) | 0.002 |
| From midlife to late adulthood | | | | | | |
|  | Stable non-obesity | 72.37 (70.25-74.49) | 62.77 (60.72-64.82) | 58.24 (56.49-59.98) | 52.05 (49.74-54.36) | <0.001 |
|  | Weight gain | 11.90 (10.54-13.26) | 16.05 (14.72-17.37) | 14.66 (13.47-15.85) | 14.00 (12.62-15.38) | 0.105 |
|  | Stable obesity | 11.03 (9.71-12.35) | 16.99 (15.04-18.95) | 21.63 (19.93-23.34) | 26.86 (24.97-28.74) | <0.001 |
|  | Weight loss | 4.70 (3.77-5.64) | 4.20 (3.49-4.90) | 5.47 (4.61-6.32) | 7.09 (5.97-8.21) | 0.001 |
| From young adulthood to late adulthood | | | | | | |
|  | Stable non-obesity | 75.26 (73.46-77.06) | 65.33 (63.21-67.46) | 62.32 (60.35-64.29) | 57.23 (54.92-59.54) | <0.001 |
|  | Weight gain | 18.51 (16.62-20.39) | 26.88 (24.95-28.81) | 29.52 (27.93-31.10) | 31.90 (29.94-33.86) | <0.001 |
|  | Stable obesity | 4.06 (2.91-5.21) | 6.30 (5.30-7.30) | 6.66 (5.71-7.61) | 9.14 (7.90-10.38) | <0.001 |
|  | Weight loss | 2.18 (1.52-2.83) | 1.49 (0.99-1.99) | 1.50 (1.11-1.89) | 1.73 (1.33-2.13) | 0.25 |
| Women | | | | | | |
| From young adulthood to midlife | | | | | | |
|  | Stable non-obesity | 85.52 (83.99-87.04) | 79.52 (78.16-80.89) | 76.46 (74.68-78.23) | 70.19 (67.89-72.48) | <0.001 |
|  | Weight gain | 10.44 (9.27-11.60) | 14.79 (13.52-16.06) | 17.38 (15.80-18.96) | 21.45 (19.66-23.25) | <0.001 |
|  | Stable obesity | 3.33 (2.53-4.14) | 4.83 (4.09-5.58) | 5.32 (4.61-6.04) | 7.43 (6.40-8.45) | <0.001 |
|  | Weight loss | 0.71 (0.32-1.10) | 0.85 (0.51-1.20) | 0.84 (0.49-1.19) | 0.93 (0.55-1.32) | 0.444 |
| From midlife to late adulthood | | | | | | |
|  | Stable non-obesity | 70.12 (67.71-72.53) | 60.87 (59.00-62.74) | 59.07 (57.01-61.14) | 52.00 (49.52-54.49) | <0.001 |
|  | Weight gain | 15.83 (14.27-17.38) | 19.43 (18.08-20.78) | 17.66 (16.44-18.87) | 19.01 (17.73-20.29) | 0.014 |
|  | Stable obesity | 11.36 (9.95-12.77) | 16.18 (14.86-17.51) | 19.57 (18.01-21.13) | 24.02 (22.06-25.98) | <0.001 |
|  | Weight loss | 2.69 (2.14-3.24) | 3.51 (3.00-4.03) | 3.70 (3.13-4.27) | 4.97 (4.12-5.82) | <0.001 |
| From young adulthood to late adulthood | | | | | | |
| Stable non-obesity | | 72.01 (69.66-74.36) | 63.38 (61.45-65.31) | 62.01 (60.05-63.98) | 55.82 (53.63-58.01) | <0.001 |
|  | Weight gain | 23.78 (21.66-25.90) | 30.82 (29.10-32.55) | 31.61 (29.85-33.38) | 35.52 (33.61-37.43) | <0.001 |
|  | Stable obesity | 3.41 (2.59-4.23) | 4.89 (4.07-5.72) | 5.55 (4.79-6.31) | 7.58 (6.55-8.61) | <0.001 |
|  | Weight loss | 0.80 (0.51-1.08) | 0.90 (0.57-1.23) | 0.83 (0.55-1.11) | 1.08 (0.73-1.43) | 0.267 |
| Mexican American | | | | | | |
| From young adulthood to midlife | | | | | | |
|  | Stable non-obesity | 79.68 (77.67-81.70) | 79.26 (76.92-81.60) | 70.36 (67.78-72.94) | 62.02 (58.78-65.25) | <0.001 |
|  | Weight gain | 14.94 (13.24-16.64) | 13.38 (11.84-14.91) | 20.72 (18.57-22.87) | 24.81 (21.83-27.79) | <0.001 |
|  | Stable obesity | 4.30 (3.58-5.03) | 6.00 (4.35-7.64) | 7.27 (5.56-8.97) | 11.97 (9.40-14.55) | <0.001 |
|  | Weight loss | 1.08 (0.53-1.63) | 1.37 (0.59-2.14) | 1.66 (0.88-2.44) | 1.19 (0.56-1.83) | 0.6 |
| From midlife to late adulthood | | | | | | |
|  | Stable non-obesity | 60.67 (58.25-63.09) | 58.96 (56.34-61.59) | 49.16 (45.40-52.92) | 41.61 (38.98-44.24) | <0.001 |
|  | Weight gain | 19.46 (17.67-21.25) | 21.33 (18.59-24.07) | 22.27 (19.37-25.17) | 20.98 (18.84-23.12) | 0.313 |
|  | Stable obesity | 14.83 (13.02-16.64) | 15.50 (13.39-17.62) | 21.96 (19.70-24.22) | 29.80 (26.70-32.90) | <0.001 |
|  | Weight loss | 5.04 (4.01-6.07) | 4.21 (3.36-5.06) | 6.61 (5.16-8.06) | 7.62 (6.17-9.07) | 0.002 |
| From young adulthood to late adulthood | | | | | | |
|  | Stable non-obesity | 65.06 (62.32-67.80) | 61.69 (58.89-64.49) | 54.28 (50.29-58.28) | 47.69 (45.25-50.13) | <0.001 |
|  | Weight gain | 29.65 (27.09-32.20) | 30.86 (28.12-33.60) | 36.96 (33.94-39.97) | 38.82 (35.76-41.87) | <0.001 |
|  | Stable obesity | 4.05 (3.06-5.04) | 5.60 (4.11-7.08) | 6.94 (5.10-8.78) | 11.83 (9.21-14.45) | <0.001 |
|  | Weight loss | 1.24 (0.72-1.76) | 1.86 (1.16-2.55) | 1.82 (1.31-2.34) | 1.66 (0.94-2.39) | 0.632 |
| Non-Hispanic Black | | | | | | |
| From young adulthood to midlife | | | | | | |
|  | Stable non-obesity | 78.87 (76.53-81.22) | 73.87 (71.15-76.58) | 66.31 (63.89-68.73) | 62.92 (60.90-64.94) | <0.001 |
|  | Weight gain | 15.15 (13.52-16.77) | 16.92 (14.98-18.86) | 21.25 (19.42-23.09) | 23.32 (21.56-25.07) | <0.001 |
|  | Stable obesity | 5.06 (3.82-6.30) | 8.01 (6.58-9.45) | 11.37 (9.79-12.94) | 12.38 (10.97-13.79) | <0.001 |
|  | Weight loss | 0.92 (0.54-1.31) | 1.20 (0.72-1.67) | 1.07 (0.52-1.62) | 1.38 (0.82-1.94) | 0.382 |
| From midlife to late adulthood | | | | | | |
|  | Stable non-obesity | 60.98 (58.39-63.57) | 51.46 (48.70-54.22) | 45.43 (43.25-47.62) | 42.21 (39.71-44.71) | <0.001 |
|  | Weight gain | 18.02 (16.42-19.63) | 23.44 (22.05-24.83) | 21.73 (20.09-23.37) | 22.18 (20.24-24.12) | 0.004 |
|  | Stable obesity | 15.12 (13.46-16.78) | 20.45 (18.28-22.61) | 27.89 (26.02-29.76) | 29.89 (27.77-32.01) | <0.001 |
|  | Weight loss | 5.87 (5.08-6.67) | 4.66 (3.81-5.50) | 4.94 (3.80-6.09) | 5.72 (4.68-6.76) | 0.675 |
| From young adulthood to late adulthood | | | | | | |
|  | Stable non-obesity | 65.61 (63.29-67.93) | 53.87 (51.19-56.54) | 48.60 (46.36-50.85) | 45.84 (43.01-48.67) | <0.001 |
|  | Weight gain | 28.21 (26.38-30.04) | 36.58 (34.60-38.56) | 38.73 (36.49-40.97) | 39.99 (37.70-42.28) | <0.001 |
|  | Stable obesity | 4.64 (3.48-5.80) | 8.01 (6.36-9.66) | 10.97 (9.13-12.81) | 12.52 (11.10-13.94) | <0.001 |
|  | Weight loss | 1.54 (1.19-1.89) | 1.54 (0.97-2.11) | 1.70 (1.16-2.23) | 1.66 (1.08-2.23) | 0.402 |
| Non-Hispanic White | | | | | | |
| From young adulthood to midlife | | | | | | |
|  | Stable non-obesity | 84.51 (82.98-86.04) | 78.16 (76.66-79.66) | 74.95 (73.07-76.82) | 66.85 (65.21-68.50) | <0.001 |
|  | Weight gain | 10.33 (9.30-11.37) | 15.61 (14.27-16.95) | 18.77 (17.25-20.29) | 24.20 (22.82-25.57) | <0.001 |
|  | Stable obesity | 4.00 (2.98-5.02) | 5.09 (4.34-5.84) | 5.46 (4.64-6.28) | 8.13 (6.79-9.47) | <0.001 |
|  | Weight loss | 1.16 (0.75-1.56) | 1.14 (0.82-1.47) | 0.83 (0.59-1.07) | 0.82 (0.46-1.18) | 0.127 |
| From midlife to late adulthood | | | | | | |
|  | Stable non-obesity | 72.59 (70.31-74.86) | 62.14 (60.47-63.82) | 60.16 (58.08-62.24) | 52.11 (50.04-54.19) | <0.001 |
|  | Weight gain | 13.08 (11.86-14.31) | 17.20 (16.13-18.27) | 15.28 (13.99-16.57) | 15.48 (14.30-16.66) | 0.025 |
|  | Stable obesity | 10.90 (9.58-12.22) | 16.76 (15.24-18.27) | 20.21 (18.60-21.82) | 26.26 (24.58-27.94) | <0.001 |
|  | Weight loss | 3.43 (2.81-4.04) | 3.90 (3.43-4.36) | 4.35 (3.67-5.03) | 6.14 (5.22-7.07) | <0.001 |
| From young adulthood to late adulthood | | | | | | |
|  | Stable non-obesity | 74.74 (72.60-76.87) | 64.93 (63.25-66.61) | 63.75 (61.74-65.76) | 56.94 (54.80-59.07) | <0.001 |
|  | Weight gain | 20.05 (18.15-21.95) | 28.59 (26.90-30.27) | 29.74 (28.01-31.48) | 33.79 (31.71-35.86) | <0.001 |
|  | Stable obesity | 3.78 (2.79-4.76) | 5.38 (4.58-6.17) | 5.56 (4.90-6.22) | 7.90 (6.67-9.13) | <0.001 |
|  | Weight loss | 1.44 (1.03-1.84) | 1.11 (0.75-1.46) | 0.95 (0.67-1.23) | 1.38 (1.02-1.74) | 0.632 |

*P* value for trend was unadjusted.

**eTable 5 Characteristics of participants presenting with weight loss, 1988-2018**

|  | 1988-1994  n=203 | 2001-2006  n=132 | 2007-2012  n=165 | 2013-2018  n=175 | *P* value for trend |
| --- | --- | --- | --- | --- | --- |
| Age, y, mean (95% CI) | 54.3 (53.1-55.5) | 54.4 (53.2-55.7) | 53.0 (51.7-54.3) | 53.4 (52.4-54.3) | <.0001 |
| Sex, % (95% CI) | | | | | |
| Men | 1.7 (1.1-2.2) | 1.4 (1.0-1.9) | 1.0 (0.7-1.4) | 0.8 (0.5-1.1) | 0.0015 |
| Race/ethnicity, % (95% CI) | | | | | |
| Mexican American | 1.1 (0.5-1.6) | 1.4 (0.6-2.1) | 1.7 (0.9-2.4) | 1.2 (0.6-1.8) | 0.6854 |
| Non-Hispanic Black | 0.9 (0.5-1.3) | 1.2 (0.7-1.7) | 1.1 (0.5-1.6) | 1.4 (0.8-1.9) | 0.2097 |
| Non-Hispanic White | 1.2 (0.8-1.6) | 1.1 (0.8-1.5) | 0.8 (0.6-1.1) | 0.8 (0.5-1.2) | 0.1354 |
| Other | 1.5 (0.0-4.0) | 0.9 (0.3-1.4) | 1.1 (0.7-1.6) | 0.5 (0.2-0.8) | 0.4723 |
| Weight, kg, mean (95% CI) | | | | | |
| At age 25 | 96.4 (93.8-99.0) | 94.4 (90.8-98.1) | 97.6 (94.0-101.2) | 100.8 (97.8-103.8) | <.0001 |
| At 10 years before survey | 87.8 (83.8-91.7) | 85.7 (83.1-88.4) | 91.9 (88.1-95.6) | 93.7 (88.3-99.1) | 0.0032 |
| At the time of survey | 79.0 (76.3-81.6) | 75.7 (72.4-79.1) | 78.0 (75.2-80.8) | 79.2 (76.4-82.0) | 0.0365 |
| Body mass index, kg/m^2^, mean (95% CI) | | | | | |
| At age 25 | 32.5 (32.0-33.0) | 32.8 (32.2-33.4) | 34.2 (33.2-35.1) | 34.7 (33.6-35.8) | <.0001 |
| At 10 years before survey | 29.6 (28.2-30.9) | 30.1 (29.0-31.1) | 31.6 (30.7-32.5) | 32.0 (30.6-33.4) | 0.0106 |
| At the time of survey | 26.4 (26.0-26.8) | 26.5 (25.9-27.0) | 26.9 (26.4-27.4) | 27.0 (26.6-27.4) | 0.0194 |
| Obesity, % (95% CI) | | | | | |
| At age 25 | 100.0 (100.0-100.0) | 100.0 (100.0-100.0) | 100.0 (100.0-100.0) | 100.0 (100.0-100.0) | 0.3167 |
| At 10 years before survey | 47.6 (34.8-60.4) | 45.1 (31.9-58.3) | 56.8 (44.7-69.0) | 65.1 (55.0-75.2) | 0.0361 |
| At the time of survey | 0.0 (0.0-0.0) | 0.0 (0.0-0.0) | 0.0 (0.0-0.0) | 0.0 (0.0-0.0) | - |
| Non-smokers, % (95% CI) | 31.1 (17.9-44.2) | 38.3 (28.6-48.0) | 44.8 (36.7-53.0) | 40.2 (31.0-49.4) | 0.2719 |

The means and percentages were weighted.

**eTable 6 Trends in the rates of weight gain (kg/year) across life course, 1988-2018**

|  | **Unweighted mean (95% CI)** | | | | |
| --- | --- | --- | --- | --- | --- |
|  | **1988-1994** | **2001-2006** | **2007-2012** | **2013-2018** | ***P* value for trend** |
| All |  |  |  |  |  |
| From young adulthood to midlife | 0.53 (0.47-0.58) | 0.54 (0.50-0.58) | 0.58 (0.55-0.62) | 0.64 (0.60-0.68) | 0.001 |
| From midlife to late adulthood | 0.13 (0.12-0.15) | 0.16 (0.15-0.17) | 0.13 (0.13-0.14) | 0.11 (0.10-0.12) | 0.001 |
| From young adulthood to late adulthood | 0.51 (0.48-0.54) | 0.59 (0.57-0.62) | 0.56 (0.55-0.58) | 0.56 (0.53-0.58) | 0.041 |
| Men |  |  |  |  |  |
| From young adulthood to midlife | 0.59 (0.50-0.67) | 0.52 (0.48-0.56) | 0.57 (0.52-0.62) | 0.62 (0.56-0.67) | 0.377 |
| From midlife to late adulthood | 0.09 (0.07-0.11) | 0.12 (0.11-0.14) | 0.11 (0.10-0.12) | 0.08 (0.07-0.09) | 0.333 |
| From young adulthood to late adulthood | 0.43 (0.38-0.47) | 0.51 (0.48-0.54) | 0.51 (0.48-0.53) | 0.49 (0.46-0.52) | 0.026 |
| Women |  |  |  |  |  |
| From young adulthood to midlife | 0.48 (0.39-0.56) | 0.56 (0.50-0.62) | 0.59 (0.55-0.64) | 0.66 (0.59-0.72) | 0.001 |
| From midlife to late adulthood | 0.18 (0.16-0.19) | 0.19 (0.18-0.21) | 0.16 (0.15-0.17) | 0.14 (0.12-0.16) | 0.001 |
| From young adulthood to late adulthood | 0.58 (0.53-0.63) | 0.66 (0.63-0.70) | 0.62 (0.60-0.64) | 0.62 (0.58-0.65) | 0.473 |
| Mexican American |  |  |  |  |  |
| From young adulthood to midlife | 0.70 (0.59-0.81) | 0.65 (0.52-0.78) | 0.79 (0.65-0.92) | 0.86 (0.75-0.98) | 0.009 |
| From midlife to late adulthood | 0.18 (0.16-0.21) | 0.21 (0.18-0.23) | 0.17 (0.14-0.20) | 0.15 (0.13-0.17) | 0.024 |
| From young adulthood to late adulthood | 0.71 (0.66-0.75) | 0.72 (0.66-0.77) | 0.70 (0.65-0.76) | 0.69 (0.65-0.73) | 0.892 |
| Non-Hispanic Black |  |  |  |  |  |
| From young adulthood to midlife | 0.72 (0.63-0.81) | 0.68 (0.59-0.77) | 0.76 (0.69-0.84) | 0.73 (0.63-0.82) | 0.482 |
| From midlife to late adulthood | 0.19 (0.17-0.21) | 0.24 (0.22-0.26) | 0.20 (0.18-0.22) | 0.19 (0.17-0.21) | 0.476 |
| From young adulthood to late adulthood | 0.72 (0.68-0.76) | 0.82 (0.78-0.86) | 0.77 (0.73-0.81) | 0.73 (0.69-0.77) | 0.896 |
| Non-Hispanic White |  |  |  |  |  |
| From young adulthood to midlife | 0.49 (0.43-0.55) | 0.52 (0.47-0.57) | 0.54 (0.49-0.59) | 0.60 (0.54-0.65) | 0.021 |
| From midlife to late adulthood | 0.12 (0.11-0.14) | 0.14 (0.13-0.16) | 0.12 (0.11-0.13) | 0.09 (0.08-0.11) | 0.003 |
| From young adulthood to late adulthood | 0.47 (0.43-0.50) | 0.55 (0.52-0.58) | 0.52 (0.50-0.55) | 0.52 (0.48-0.55) | 0.06 |

P value for trend were unadjusted.

**eTable 7 Trends in the rates of weight gain (kg/year) across life course stratified by age at the time of the survey, 1988-2018**

|  | Survey-Weighted mean (95% CI) | | | | |
| --- | --- | --- | --- | --- | --- |
|  | 1988-1994 | 2001-2006 | 2007-2012 | 2013-2018 | *P* value for trend |
| 35-49 years at the time of the survey | | | | | |
| All^a^ |  |  |  |  |  |
| From young adulthood to midlife | 0.75 (0.64-0.86) | 0.70 (0.61-0.80) | 0.83 (0.73-0.92) | 0.96 (0.84-1.08) | 0.0057 |
| From midlife to late adulthood | 0.23 (0.21-0.25) | 0.26 (0.24-0.28) | 0.24 (0.22-0.25) | 0.24 (0.21-0.26) | 0.6855 |
| From young adulthood to late adulthood | 0.70 (0.65-0.75) | 0.80 (0.75-0.84) | 0.78 (0.74-0.81) | 0.81 (0.76-0.85) | 0.0226 |
| Men^b^ |  |  |  |  |  |
| From young adulthood to midlife | 0.87 (0.69-1.06) | 0.68 (0.58-0.77) | 0.83 (0.69-0.96) | 0.96 (0.79-1.12) | 0.3516 |
| From midlife to late adulthood | 0.16 (0.12-0.20) | 0.21 (0.19-0.23) | 0.20 (0.18-0.22) | 0.20 (0.17-0.23) | 0.2770 |
| From young adulthood to late adulthood | 0.58 (0.49-0.66) | 0.68 (0.63-0.73) | 0.69 (0.65-0.74) | 0.72 (0.65-0.78) | 0.0219 |
| Women^b^ |  |  |  |  |  |
| From young adulthood to midlife | 0.64 (0.46-0.81) | 0.73 (0.58-0.88) | 0.83 (0.70-0.95) | 0.97 (0.77-1.17) | 0.0127 |
| From midlife to late adulthood | 0.30 (0.27-0.33) | 0.31 (0.29-0.34) | 0.28 (0.25-0.30) | 0.27 (0.24-0.31) | 0.0870 |
| From young adulthood to late adulthood | 0.82 (0.74-0.89) | 0.91 (0.86-0.97) | 0.86 (0.81-0.91) | 0.89 (0.82-0.96) | 0.5022 |
| 50-64 years at the time of the survey | | | | | |
| All^a^ |  |  |  |  |  |
| From young adulthood to midlife | 0.44 (0.40-0.47) | 0.49 (0.46-0.53) | 0.51 (0.48-0.54) | 0.58 (0.53-0.62) | <.0001 |
| From midlife to late adulthood | 0.10 (0.09-0.12) | 0.13 (0.11-0.14) | 0.11 (0.10-0.12) | 0.09 (0.07-0.10) | 0.1175 |
| From young adulthood to late adulthood | 0.45 (0.42-0.47) | 0.53 (0.50-0.55) | 0.52 (0.49-0.54) | 0.52 (0.49-0.55) | 0.0003 |
| Men^b^ |  |  |  |  |  |
| From young adulthood to midlife | 0.41 (0.36-0.46) | 0.46 (0.43-0.50) | 0.48 (0.44-0.52) | 0.54 (0.50-0.58) | <.0001 |
| From midlife to late adulthood | 0.05 (0.03-0.07) | 0.09 (0.07-0.10) | 0.08 (0.07-0.10) | 0.05 (0.04-0.07) | 0.9022 |
| From young adulthood to late adulthood | 0.36 (0.32-0.39) | 0.44 (0.41-0.48) | 0.45 (0.42-0.48) | 0.45 (0.42-0.48) | 0.0002 |
| Women^b^ |  |  |  |  |  |
| From young adulthood to midlife | 0.46 (0.41-0.50) | 0.52 (0.47-0.57) | 0.54 (0.50-0.58) | 0.61 (0.54-0.68) | 0.0001 |
| From midlife to late adulthood | 0.15 (0.13-0.17) | 0.17 (0.14-0.19) | 0.14 (0.12-0.15) | 0.12 (0.09-0.15) | 0.0562 |
| From young adulthood to late adulthood | 0.53 (0.49-0.57) | 0.60 (0.57-0.64) | 0.58 (0.55-0.61) | 0.59 (0.55-0.63) | 0.0319 |
| ≥65 years at the time of the survey | | | | | |
| All^a^ |  |  |  |  |  |
| From young adulthood to midlife | 0.24 (0.23-0.25) | 0.29 (0.27-0.30) | 0.32 (0.30-0.33) | 0.36 (0.34-0.37) | <.0001 |
| From midlife to late adulthood | 0.00 (-0.00-0.01) | 0.02 (0.02-0.03) | 0.02 (0.01-0.03) | 0.00 (-0.01-0.01) | 0.2795 |
| From young adulthood to late adulthood | 0.20 (0.18-0.21) | 0.26 (0.25-0.28) | 0.28 (0.27-0.30) | 0.28 (0.27-0.30) | <.0001 |
| Men^b^ |  |  |  |  |  |
| From young adulthood to midlife | 0.25 (0.23-0.27) | 0.27 (0.25-0.29) | 0.31 (0.29-0.32) | 0.33 (0.31-0.36) | <.0001 |
| From midlife to late adulthood | -0.02 (-0.03--0.01) | 0.00 (-0.01-0.01) | 0.00 (-0.01-0.01) | -0.02 (-0.03--0.01) | 0.5838 |
| From young adulthood to late adulthood | 0.17 (0.15-0.19) | 0.22 (0.20-0.23) | 0.25 (0.23-0.27) | 0.23 (0.22-0.25) | <.0001 |
| Women^b^ |  |  |  |  |  |
| From young adulthood to midlife | 0.23 (0.21-0.25) | 0.30 (0.28-0.32) | 0.33 (0.31-0.35) | 0.38 (0.36-0.40) | <.0001 |
| From midlife to late adulthood | 0.02 (0.01-0.03) | 0.04 (0.03-0.05) | 0.03 (0.02-0.05) | 0.02 (0.01-0.03) | 0.2427 |
| From young adulthood to late adulthood | 0.22 (0.20-0.23) | 0.30 (0.28-0.32) | 0.31 (0.29-0.34) | 0.32 (0.31-0.34) | <.0001 |

^a^*P* value for trend was adjusted for age, sex, and race/ethnicity;

^b^*P* value for trend was adjusted for age and race/ethnicity.

**eTable 8 Trends in weight change patterns across life course among participants aged 45 years or older at the time of survey, 1988-2018**

|  | Survey-weighted percentage (95% CI) | | | | |
| --- | --- | --- | --- | --- | --- |
|  | 1988-1994 | 2001-2006 | 2007-2012 | 2013-2018 | *P* value for trend |
| *All* ^a^ | | | | | |
| From young adulthood to midlife | | | | | |
| Stable non-obesity | 82.1 (80.8-83.4) | 76.8 (75.5-78.2) | 72.7 (70.9-74.6) | 66.8 (65.0-68.6) | <.0001 |
| Weight gain | 13.3 (12.3-14.2) | 18.2 (16.9-19.5) | 21.9 (20.5-23.4) | 25.7 (24.1-27.3) | <.0001 |
| Stable obesity | 3.5 (2.5-4.4) | 3.9 (3.5-4.4) | 4.5 (3.8-5.2) | 6.7 (5.6-7.8) | <.0001 |
| Weight loss | 1.2 (0.8-1.5) | 1.0 (0.7-1.3) | 0.8 (0.6-1.0) | 0.8 (0.5-1.1) | 0.0217 |
| From midlife to late adulthood | | | | | |
| Stable non-obesity | 69.5 (67.6-71.3) | 61.4 (59.8-63.0) | 58.3 (56.4-60.2) | 52.4 (50.4-54.4) | <.0001 |
| Weight gain | 13.7 (12.5-15.0) | 16.5 (15.6-17.4) | 15.0 (14.1-15.9) | 15.2 (14.1-16.3) | 0.2558 |
| Stable obesity | 12.3 (11.2-13.5) | 17.7 (16.3-19.1) | 21.6 (20.0-23.2) | 26.0 (24.2-27.8) | <.0001 |
| Weight loss | 4.5 (3.9-5.1) | 4.4 (3.9-4.9) | 5.1 (4.5-5.8) | 6.4 (5.6-7.1) | <.0001 |
| From young adulthood to late adulthood | | | | | |
| Stable non-obesity | 72.3 (70.4-74.2) | 64.4 (62.9-66.0) | 62.3 (60.5-64.1) | 57.4 (55.4-59.4) | <.0001 |
| Weight gain | 23.1 (21.4-24.9) | 30.7 (29.1-32.3) | 32.4 (30.9-33.9) | 35.0 (33.2-36.8) | <.0001 |
| Stable obesity | 3.1 (2.3-4.0) | 3.8 (3.3-4.3) | 4.4 (3.7-5.0) | 6.5 (5.4-7.5) | <.0001 |
| Weight loss | 1.5 (1.1-1.8) | 1.0 (0.8-1.3) | 0.9 (0.7-1.2) | 1.1 (0.8-1.4) | 0.0312 |
| Men ^b^ |  |  |  |  |  |
| From young adulthood to midlife |  |  |  |  |  |
| Stable non-obesity | 80.4 (78.4-82.3) | 75.3 (73.1-77.5) | 70.5 (68.3-72.7) | 64.1 (62.1-66.2) | <.0001 |
| Weight gain | 14.1 (12.5-15.6) | 19.0 (17.0-20.9) | 23.5 (21.7-25.3) | 27.7 (25.7-29.7) | <.0001 |
| Stable obesity | 3.9 (2.5-5.3) | 4.5 (3.8-5.2) | 5.0 (4.0-6.1) | 7.5 (6.1-8.9) | <.0001 |
| Weight loss | 1.7 (1.0-2.4) | 1.2 (0.7-1.7) | 1.0 (0.6-1.4) | 0.7 (0.4-1.0) | 0.0075 |
| From midlife to late adulthood |  |  |  |  |  |
| Stable non-obesity | 70.9 (68.5-73.3) | 62.6 (60.4-64.7) | 58.2 (56.1-60.3) | 52.8 (50.5-55.0) | <.0001 |
| Weight gain | 11.4 (9.9-13.0) | 14.1 (12.7-15.5) | 13.3 (12.2-14.5) | 12.1 (10.6-13.5) | 0.7639 |
| Stable obesity | 11.7 (10.1-13.4) | 18.2 (16.0-20.5) | 22.3 (20.4-24.3) | 27.6 (25.4-29.9) | <.0001 |
| Weight loss | 5.9 (4.9-7.0) | 5.1 (4.3-5.9) | 6.2 (5.1-7.2) | 7.5 (6.3-8.8) | 0.0162 |
| From young adulthood to late adulthood | | | | | |
| Stable non-obesity | 74.2 (72.0-76.5) | 66.2 (63.9-68.6) | 63.0 (60.8-65.3) | 58.5 (56.0-61.0) | <.0001 |
| Weight gain | 20.2 (18.0-22.5) | 28.0 (25.8-30.3) | 31.0 (29.1-32.9) | 33.3 (31.2-35.3) | <.0001 |
| Stable obesity | 3.2 (2.1-4.4) | 4.4 (3.6-5.1) | 4.8 (3.8-5.8) | 6.8 (5.5-8.2) | <.0001 |
| Weight loss | 2.3 (1.5-3.1) | 1.4 (0.9-1.8) | 1.2 (0.8-1.6) | 1.4 (1.0-1.8) | 0.0514 |
| Women^b^ |  |  |  |  |  |
| From young adulthood to midlife |  |  |  |  |  |
| Stable non-obesity | 83.6 (81.8-85.3) | 78.2 (76.6-79.7) | 74.9 (72.6-77.1) | 69.1 (66.4-71.9) | <.0001 |
| Weight gain | 12.6 (11.3-13.9) | 17.6 (16.0-19.1) | 20.5 (18.5-22.6) | 23.9 (21.5-26.3) | <.0001 |
| Stable obesity | 3.1 (2.2-4.0) | 3.5 (2.8-4.1) | 4.0 (3.3-4.7) | 6.0 (4.8-7.2) | 0.0002 |
| Weight loss | 0.7 (0.3-1.1) | 0.8 (0.4-1.2) | 0.6 (0.3-0.9) | 0.9 (0.5-1.4) | 0.9493 |
| From midlife to late adulthood |  |  |  |  |  |
| Stable non-obesity | 68.1 (65.8-70.4) | 60.2 (58.2-62.2) | 58.4 (55.7-61.1) | 52.1 (49.0-55.2) | <.0001 |
| Weight gain | 15.8 (14.1-17.5) | 18.7 (17.5-20.0) | 16.5 (15.1-17.8) | 18.1 (16.5-19.6) | 0.1923 |
| Stable obesity | 12.9 (11.4-14.3) | 17.3 (15.7-18.8) | 20.9 (18.9-22.9) | 24.6 (22.2-27.0) | <.0001 |
| Weight loss | 3.2 (2.5-3.9) | 3.8 (3.1-4.4) | 4.2 (3.5-4.9) | 5.3 (4.4-6.2) | 0.0005 |
| From young adulthood to late adulthood | | | | | |
| Stable non-obesity | 70.4 (68.2-72.6) | 62.8 (60.8-64.8) | 61.6 (59.2-64.1) | 56.4 (53.7-59.1) | <.0001 |
| Weight gain | 25.8 (23.8-27.7) | 33.2 (31.2-35.2) | 33.7 (31.4-36.0) | 36.6 (34.1-39.1) | <.0001 |
| Stable obesity | 3.0 (2.1-3.9) | 3.3 (2.6-4.0) | 3.9 (3.3-4.6) | 6.2 (5.0-7.3) | <.0001 |
| Weight loss | 0.8 (0.4-1.1) | 0.7 (0.5-1.0) | 0.7 (0.4-1.0) | 0.9 (0.5-1.3) | 0.7383 |

^a^*P* value for trend was adjusted for age, sex, and race/ethnicity;

^b^*P* value for trend was adjusted for age and race/ethnicity.

**eTable 9 Trends in the rates of weight gain (kg/year) across life course among participants aged 45 or older at the time of survey, 1988-2018**

|  | Survey-Weighted mean (95% CI) | | | | |
| --- | --- | --- | --- | --- | --- |
|  | 1988-1994 | 2001-2006 | 2007-2012 | 2013-2018 | *P* value for trend |
| All^a^ |  |  |  |  |  |
| From young adulthood to midlife | 0.38 (0.36- 0.41) | 0.45 (0.43- 0.48) | 0.47 (0.45- 0.49) | 0.53 (0.49- 0.56) | <.0001 |
| From midlife to late adulthood | 0.08 (0.07- 0.09) | 0.11 (0.10- 0.12) | 0.09 (0.08- 0.10) | 0.08 (0.07- 0.09) | 0.0621 |
| From young adulthood to late adulthood | 0.38 (0.36- 0.40) | 0.47 (0.45- 0.49) | 0.46 (0.44- 0.47) | 0.48 (0.45- 0.50) | <.0001 |
| Men^b^ |  |  |  |  |  |
| From young adulthood to midlife | 0.38 (0.35- 0.41) | 0.42 (0.39- 0.45) | 0.44 (0.41- 0.47) | 0.49 (0.47- 0.52) | <.0001 |
| From midlife to late adulthood | 0.04 (0.02- 0.05) | 0.07 (0.06- 0.08) | 0.06 (0.06- 0.07) | 0.05 (0.04- 0.06) | 0.5450 |
| From young adulthood to late adulthood | 0.31 (0.28- 0.34) | 0.39 (0.37- 0.42) | 0.40 (0.37- 0.42) | 0.41 (0.39- 0.43) | <.0001 |
| Women^b^ |  |  |  |  |  |
| From young adulthood to midlife | 0.38 (0.34- 0.42) | 0.48 (0.46- 0.51) | 0.50 (0.48- 0.53) | 0.56 (0.51- 0.61) | <.0001 |
| From midlife to late adulthood | 0.12 (0.11- 0.14) | 0.14 (0.13- 0.15) | 0.11 (0.11- 0.12) | 0.10 (0.09- 0.12) | 0.0021 |
| From young adulthood to late adulthood | 0.45 (0.42- 0.47) | 0.54 (0.52- 0.56) | 0.52 (0.50- 0.54) | 0.54 (0.50- 0.57) | 0.0002 |
| Mexican American^c^ |  |  |  |  |  |
| From young adulthood to midlife | 0.51 (0.46- 0.56) | 0.47 (0.43- 0.52) | 0.54 (0.49- 0.60) | 0.57 (0.55- 0.58) | 0.0027 |
| From midlife to late adulthood | 0.07 (0.05- 0.08) | 0.09 (0.07- 0.11) | 0.06 (0.04- 0.08) | 0.07 (0.06- 0.09) | 0.7057 |
| From young adulthood to late adulthood | 0.46 (0.42- 0.49) | 0.46 (0.42- 0.49) | 0.46 (0.42- 0.51) | 0.50 (0.49- 0.52) | 0.0230 |
| Non-Hispanic Black^c^ |  |  |  |  |  |
| From young adulthood to midlife | 0.49 (0.46- 0.52) | 0.55 (0.51- 0.58) | 0.56 (0.52- 0.60) | 0.56 (0.52- 0.61) | 0.0043 |
| From midlife to late adulthood | 0.09 (0.07- 0.11) | 0.14 (0.12- 0.15) | 0.12 (0.10- 0.14) | 0.12 (0.10- 0.13) | 0.0433 |
| From young adulthood to late adulthood | 0.48 (0.45- 0.51) | 0.59 (0.56- 0.62) | 0.57 (0.54- 0.59) | 0.57 (0.54- 0.60) | <.0001 |
| Non-Hispanic White^c^ |  |  |  |  |  |
| From young adulthood to midlife | 0.36 (0.33- 0.39) | 0.44 (0.42- 0.47) | 0.45 (0.43- 0.48) | 0.52 (0.48- 0.56) | <.0001 |
| From midlife to late adulthood | 0.08 (0.07- 0.09) | 0.11 (0.10- 0.12) | 0.09 (0.08- 0.10) | 0.07 (0.06- 0.09) | 0.0413 |
| From young adulthood to late adulthood | 0.37 (0.35- 0.39) | 0.46 (0.44- 0.48) | 0.45 (0.42- 0.47) | 0.47 (0.44- 0.50) | <.0001 |

^a^*P* value for trend was adjusted for age, sex, and race/ethnicity;

^b^*P* value for trend was adjusted for age and race/ethnicity;

^c^*P* value for trend was adjusted for age and sex.

**eTable 10 Trends in weight change patterns across life course among participants aged between 45 and 65 years at the time of survey, 1988-2018**

|  | Survey-weighted percentage (95% CI) | | | | |
| --- | --- | --- | --- | --- | --- |
|  | 1988-1994 | 2001-2006 | 2007-2012 | 2013-2018 | *P* value for trend |
| All^a^ |  |  |  |  |  |
| From young adulthood to midlife | | | | | |
| Stable non-obesity | 85.5 (84.1-86.8) | 79.5 (77.9-81.2) | 75.9 (74.5-77.4) | 70.6 (68.7-72.6) | <.0001 |
| Weight gain | 9.2 (8.4-10.1) | 12.9 (11.6-14.2) | 15.2 (14.1-16.3) | 17.0 (15.7-18.4) | <.0001 |
| Stable obesity | 4.3 (3.4- 5.3) | 6.3 (5.4- 7.2) | 7.7 (6.9- 8.6) | 11.3 (10.0-12.5) | <.0001 |
| Weight loss | 1.0 (0.6- 1.3) | 1.3 (0.9- 1.6) | 1.1 (0.8- 1.4) | 1.1 (0.7- 1.5) | 0.9528 |
| From midlife to late adulthood | | | | | |
| Stable non-obesity | 70.8 (68.5-73.1) | 61.1 (59.1-63.1) | 58.7 (57.2-60.2) | 52.4 (50.0-54.8) | <.0001 |
| Weight gain | 15.7 (14.3-17.1) | 19.7 (18.5-21.0) | 18.0 (16.8-19.1) | 19.2 (18.0-20.4) | 0.0235 |
| Stable obesity | 10.7 (9.5-11.9) | 16.1 (14.4-17.7) | 19.6 (18.3-20.9) | 24.0 (22.2-25.7) | <.0001 |
| Weight loss | 2.8 (2.1- 3.5) | 3.1 (2.7- 3.6) | 3.7 (3.1- 4.3) | 4.5 (3.8- 5.1) | 0.0003 |
| From young adulthood to late adulthood | | | | | |
| Stable non-obesity | 72.6 (70.5-74.7) | 63.1 (61.0-65.1) | 61.5 (59.7-63.2) | 55.2 (53.0-57.3) | <.0001 |
| Weight gain | 22.0 (20.2-23.9) | 29.1 (27.3-30.8) | 29.5 (28.1-30.9) | 32.2 (30.4-33.9) | <.0001 |
| Stable obesity | 4.1 (3.2- 5.0) | 6.7 (5.8- 7.6) | 7.7 (6.8- 8.5) | 10.9 (9.7-12.1) | <.0001 |
| Weight loss | 1.2 (0.8- 1.6) | 1.2 (0.8- 1.5) | 1.4 (1.1- 1.7) | 1.8 (1.4- 2.2) | 0.0424 |
| Men^b^ |  |  |  |  |  |
| From young adulthood to midlife |  |  |  |  |  |
| Stable non-obesity | 84.0 (82.1-86.0) | 78.3 (76.2-80.4) | 73.3 (71.2-75.4) | 68.5 (66.2-70.8) | <.0001 |
| Weight gain | 9.6 (8.2-11.0) | 13.4 (11.8-15.0) | 16.9 (15.4-18.4) | 18.1 (16.5-19.7) | <.0001 |
| Stable obesity | 5.0 (3.7- 6.3) | 6.7 (5.5- 7.9) | 8.7 (7.3-10.0) | 12.5 (10.7-14.2) | <.0001 |
| Weight loss | 1.4 (0.8- 2.0) | 1.6 (1.0- 2.2) | 1.2 (0.8- 1.6) | 0.9 (0.5- 1.4) | 0.1135 |
| From midlife to late adulthood |  |  |  |  |  |
| Stable non-obesity | 72.4 (70.0-74.9) | 62.1 (59.5-64.7) | 58.0 (55.9-60.0) | 52.3 (49.3-55.2) | <.0001 |
| Weight gain | 13.1 (11.5-14.7) | 17.9 (16.2-19.6) | 16.3 (14.8-17.8) | 17.1 (15.1-19.1) | 0.0634 |
| Stable obesity | 10.6 (9.1-12.0) | 16.6 (14.6-18.6) | 21.0 (19.1-23.0) | 25.2 (23.1-27.3) | <.0001 |
| Weight loss | 3.9 (2.6- 5.2) | 3.4 (2.6- 4.2) | 4.7 (3.7- 5.7) | 5.5 (4.4- 6.6) | 0.0193 |
| From young adulthood to late adulthood | | | | | |
| Stable non-obesity | 75.0 (72.9-77.1) | 64.0 (61.4-66.6) | 61.3 (59.0-63.6) | 55.8 (53.0-58.6) | <.0001 |
| Weight gain | 18.7 (16.6-20.8) | 27.2 (24.9-29.5) | 28.7 (26.9-30.5) | 30.4 (28.1-32.7) | <.0001 |
| Stable obesity | 4.4 (3.1- 5.7) | 7.3 (6.1- 8.6) | 8.2 (6.9- 9.5) | 11.7 (10.0-13.4) | <.0001 |
| Weight loss | 1.9 (1.1- 2.7) | 1.5 (0.8- 2.1) | 1.8 (1.3- 2.3) | 2.0 (1.4- 2.7) | 0.6647 |
| Women ^b^ |  |  |  |  |  |
| From young adulthood to midlife |  |  |  |  |  |
| Stable non-obesity | 86.8 (85.2-88.4) | 80.7 (79.0-82.4) | 78.5 (76.7-80.3) | 72.6 (69.9-75.4) | <.0001 |
| Weight gain | 8.9 (7.7-10.2) | 12.4 (10.9-13.9) | 13.6 (12.2-15.0) | 16.0 (14.0-18.0) | <.0001 |
| Stable obesity | 3.7 (2.7- 4.6) | 6.0 (5.0- 7.0) | 6.9 (5.8- 7.9) | 10.1 (8.8-11.5) | <.0001 |
| Weight loss | 0.6 (0.2- 0.9) | 0.9 (0.5- 1.3) | 1.0 (0.5- 1.6) | 1.2 (0.7- 1.7) | 0.0630 |
| From midlife to late adulthood |  |  |  |  |  |
| Stable non-obesity | 69.2 (66.4-72.1) | 60.1 (57.8-62.4) | 59.4 (57.3-61.6) | 52.5 (49.6-55.4) | <.0001 |
| Weight gain | 18.1 (16.1-20.2) | 21.5 (19.8-23.3) | 19.6 (18.1-21.1) | 21.2 (19.6-22.8) | 0.1388 |
| Stable obesity | 10.9 (9.3-12.4) | 15.5 (13.8-17.3) | 18.2 (16.6-19.7) | 22.8 (20.4-25.1) | <.0001 |
| Weight loss | 1.8 (1.2- 2.4) | 2.9 (2.2- 3.5) | 2.8 (2.3- 3.4) | 3.5 (2.6- 4.4) | 0.0053 |
| From young adulthood to late adulthood | | | | | |
| Stable non-obesity | 70.4 (67.5-73.2) | 62.2 (59.7-64.6) | 61.7 (59.4-63.9) | 54.6 (51.9-57.2) | <.0001 |
| Weight gain | 25.1 (22.5-27.8) | 30.9 (28.9-32.9) | 30.2 (28.4-32.1) | 33.8 (31.6-36.0) | <.0001 |
| Stable obesity | 3.9 (2.9- 4.8) | 6.1 (5.0- 7.2) | 7.1 (6.0- 8.2) | 10.1 (8.8-11.5) | <.0001 |
| Weight loss | 0.6 (0.3- 0.9) | 0.9 (0.4- 1.3) | 1.0 (0.6- 1.4) | 1.5 (1.0- 2.0) | 0.0041 |

^a^*P* value for trend was adjusted for age, sex, and race/ethnicity;

^b^*P* value for trend was adjusted for age and race/ethnicity.

**eTable 11 Trends in the rates of weight gain (kg/year) across life course among participants aged between 45 and 65 years at the time of survey, 1988-2018**

|  | Survey-Weighted mean (95% CI) | | | | |
| --- | --- | --- | --- | --- | --- |
|  | 1988-1994 | 2001-2006 | 2007-2012 | 2013-2018 | *P* value for trend |
| All^a^ |  |  |  |  |  |
| From young adulthood to midlife | 0.62 (0.55-0.69) | 0.63 (0.57-0.68) | 0.70 (0.64-0.76) | 0.80 (0.73-0.87) | 0.0001 |
| From midlife to late adulthood | 0.18 (0.16-0.19) | 0.21 (0.19-0.22) | 0.18 (0.17-0.20) | 0.18 (0.16-0.19) | 0.1989 |
| From young adulthood to late adulthood | 0.60 (0.57-0.63) | 0.69 (0.66-0.72) | 0.67 (0.65-0.70) | 0.69 (0.66-0.72) | 0.0006 |
| Men^b^ |  |  |  |  |  |
| From young adulthood to midlife | 0.68 (0.58-0.79) | 0.60 (0.54-0.65) | 0.69 (0.61-0.76) | 0.78 (0.69-0.87) | 0.1055 |
| From midlife to late adulthood | 0.12 (0.09-0.14) | 0.16 (0.15-0.18) | 0.15 (0.14-0.16) | 0.14 (0.12-0.16) | 0.3916 |
| From young adulthood to late adulthood | 0.49 (0.43-0.55) | 0.59 (0.55-0.62) | 0.60 (0.57-0.63) | 0.61 (0.57-0.65) | 0.0010 |
| Women^b^ |  |  |  |  |  |
| From young adulthood to midlife | 0.56 (0.45-0.67) | 0.66 (0.57-0.74) | 0.71 (0.64-0.79) | 0.82 (0.71-0.94) | 0.0011 |
| From midlife to late adulthood | 0.24 (0.21-0.26) | 0.25 (0.23-0.27) | 0.22 (0.20-0.23) | 0.21 (0.18-0.24) | 0.0198 |
| From young adulthood to late adulthood | 0.70 (0.65-0.76) | 0.79 (0.75-0.82) | 0.75 (0.71-0.78) | 0.77 (0.72-0.81) | 0.1731 |
| Mexican American^c^ |  |  |  |  |  |
| From young adulthood to midlife | 0.71 (0.71-0.72) | 0.67 (0.65-0.70) | 0.81 (0.67-0.96) | 0.94 (0.94-0.94) | <.0001 |
| From midlife to late adulthood | 0.20 (0.20-0.20) | 0.22 (0.21-0.23) | 0.19 (0.16-0.22) | 0.17 (0.15-0.20) | <.0001 |
| From young adulthood to late adulthood | 0.71 (0.69-0.74) | 0.73 (0.72-0.74) | 0.72 (0.67-0.78) | 0.74 (0.74-0.74) | <.0001 |
| Non-Hispanic Black^c^ |  |  |  |  |  |
| From young adulthood to midlife | 0.79 (0.69-0.89) | 0.75 (0.63-0.88) | 0.90 (0.79-1.01) | 0.87 (0.72-1.01) | 0.1864 |
| From midlife to late adulthood | 0.24 (0.21-0.26) | 0.29 (0.27-0.31) | 0.27 (0.24-0.29) | 0.26 (0.23-0.29) | 0.3173 |
| From young adulthood to late adulthood | 0.79 (0.76-0.83) | 0.92 (0.87-0.97) | 0.89 (0.84-0.95) | 0.87 (0.81-0.93) | 0.0254 |
| Non-Hispanic White^c^ |  |  |  |  |  |
| From young adulthood to midlife | 0.59 (0.51-0.67) | 0.62 (0.54-0.69) | 0.67 (0.59-0.75) | 0.77 (0.66-0.89) | 0.0023 |
| From midlife to late adulthood | 0.17 (0.15-0.19) | 0.19 (0.17-0.21) | 0.17 (0.15-0.19) | 0.16 (0.14-0.19) | 0.3192 |
| From young adulthood to late adulthood | 0.56 (0.52-0.60) | 0.65 (0.62-0.69) | 0.64 (0.60-0.67) | 0.66 (0.61-0.70) | 0.0023 |

^a^*P* value for trend was adjusted for age, sex, and race/ethnicity;

^b^*P* value for trend was adjusted for age and race/ethnicity;

^c^*P* value for trend was adjusted for age and sex.

**eTable 12 Trends in weight change patterns across life course among participants aged≥ 65 years at the time of survey, 1988-2018**

|  | Survey-weighted percentage (95% CI) | | | | |
| --- | --- | --- | --- | --- | --- |
|  | 1988-1994 | 2001-2006 | 2007-2012 | 2013-2018 | *P* value for trend |
| All^a^ |  |  |  |  |  |
| From young adulthood to midlife | | | | | |
| Stable non-obesity | 80.3 (78.1-82.5) | 75.6 (73.9-77.4) | 70.6 (68.2-73.0) | 63.4 (60.7-66.1) | <.0001 |
| Weight gain | 15.3 (13.5-17.1) | 21.0 (19.3-22.7) | 26.5 (24.3-28.7) | 33.1 (30.5-35.7) | <.0001 |
| Stable obesity | 2.9 (1.9-3.8) | 2.5 (2.1-2.9) | 2.4 (1.9-2.8) | 3.0 (2.2-3.8) | 0.6952 |
| Weight loss | 1.6 (1.1-2.0) | 0.8 (0.5-1.2) | 0.6 (0.3-0.9) | 0.5 (0.2-0.8) | <.0001 |
| From midlife to late adulthood | | | | | |
| Stable non-obesity | 72.7 (70.3-75.0) | 65.1 (63.2-67.1) | 59.9 (57.6-62.3) | 52.7 (50.3-55.0) | <.0001 |
| Weight gain | 8.8 (7.6-10.1) | 11.3 (9.8-12.8) | 11.1 (9.7-12.5) | 11.4 (9.9-12.9) | 0.0314 |
| Stable obesity | 12.2 (10.4-14.0) | 17.3 (15.8-18.9) | 22.1 (20.0-24.2) | 26.6 (24.2-29.0) | <.0001 |
| Weight loss | 6.3 (5.2-7.4) | 6.3 (5.4-7.1) | 6.9 (5.8-8.0) | 9.3 (8.0-10.7) | 0.0004 |
| From young adulthood to late adulthood | | | | | |
| Stable non-obesity | 76.7 (74.6-78.8) | 69.6 (67.6-71.6) | 65.7 (63.6-67.8) | 61.2 (58.6-63.7) | <.0001 |
| Weight gain | 19.0 (17.1-20.8) | 27.1 (25.2-29.1) | 31.4 (29.3-33.4) | 35.3 (32.8-37.8) | <.0001 |
| Stable obesity | 2.3 (1.4-3.2) | 1.9 (1.5-2.3) | 2.4 (1.8-3.0) | 2.8 (2.0-3.6) | 0.2534 |
| Weight loss | 2.0 (1.5-2.5) | 1.4 (0.9-1.8) | 0.5 (0.3-0.7) | 0.7 (0.5-1.0) | <.0001 |
| Men^b^ |  |  |  |  |  |
| From young adulthood to midlife |  |  |  |  |  |
| Stable non-obesity | 78.2 (75.9-80.6) | 74.0 (71.1-76.9) | 68.6 (66.2-71.1) | 60.6 (57.1-64.2) | <.0001 |
| Weight gain | 15.8 (13.6-18.0) | 21.8 (19.1-24.5) | 28.2 (25.6-30.8) | 34.7 (31.1-38.3) | <.0001 |
| Stable obesity | 3.5 (2.0-5.0) | 3.2 (2.3-4.1) | 2.6 (1.8-3.5) | 3.9 (2.7-5.1) | 0.4310 |
| Weight loss | 2.5 (1.6-3.3) | 1.0 (0.4-1.5) | 0.6 (0.1-1.0) | 0.7 (0.2-1.2) | 0.0003 |
| From midlife to late adulthood |  |  |  |  |  |
| Stable non-obesity | 73.3 (70.1-76.5) | 65.8 (63.4-68.2) | 60.3 (57.2-63.4) | 53.5 (50.3-56.6) | <.0001 |
| Weight gain | 7.7 (5.9-9.5) | 9.4 (7.6-11.2) | 9.2 (7.6-10.7) | 8.2 (6.6-9.8) | 0.8890 |
| Stable obesity | 11.3 (8.8-13.9) | 17.5 (14.7-20.3) | 22.6 (20.5-24.8) | 28.0 (24.3-31.8) | <.0001 |
| Weight loss | 7.7 (6.1-9.3) | 7.3 (5.6-8.9) | 7.9 (6.4-9.4) | 10.3 (8.0-12.5) | 0.0498 |
| From young adulthood to late adulthood | | | | | |
| Stable non-obesity | 77.4 (74.2-80.6) | 71.2 (68.1-74.2) | 67.3 (64.4-70.2) | 62.4 (58.7-66.1) | <.0001 |
| Weight gain | 16.7 (13.9-19.6) | 24.7 (21.8-27.7) | 29.5 (26.4-32.6) | 32.9 (29.3-36.5) | <.0001 |
| Stable obesity | 2.8 (1.4-4.2) | 2.5 (1.7-3.2) | 2.6 (1.6-3.5) | 3.5 (2.3-4.8) | 0.2296 |
| Weight loss | 3.1 (2.2-4.0) | 1.6 (0.9-2.3) | 0.6 (0.2-1.0) | 1.1 (0.6-1.7) | <.0001 |
| Women ^b^ |  |  |  |  |  |
| From young adulthood to midlife |  |  |  |  |  |
| Stable non-obesity | 82.0 (79.3-84.7) | 76.8 (74.1-79.6) | 72.2 (68.3-76.0) | 65.8 (62.6-69.1) | <.0001 |
| Weight gain | 14.7 (12.5-16.8) | 20.5 (17.8-23.3) | 25.2 (21.6-28.8) | 31.6 (28.6-34.6) | <.0001 |
| Stable obesity | 2.4 (1.4-3.4) | 1.9 (1.3-2.5) | 2.1 (1.4-2.8) | 2.2 (1.3-3.2) | 0.8152 |
| Weight loss | 0.9 (0.3-1.5) | 0.7 (0.3-1.1) | 0.6 (0.2-0.9) | 0.4 (0.0-0.7) | 0.0497 |
| From midlife to late adulthood |  |  |  |  |  |
| Stable non-obesity | 72.3 (69.3-75.2) | 64.5 (61.6-67.3) | 59.6 (56.0-63.3) | 52.2 (49.2-55.2) | <.0001 |
| Weight gain | 9.8 (7.9-11.6) | 12.8 (10.9-14.7) | 12.7 (10.9-14.4) | 14.0 (11.8-16.2) | 0.0153 |
| Stable obesity | 12.7 (10.3-15.1) | 17.2 (14.6-19.9) | 21.6 (18.5-24.7) | 25.3 (22.6-28.0) | <.0001 |
| Weight loss | 5.3 (4.1-6.4) | 5.5 (4.4-6.6) | 6.1 (4.6-7.6) | 8.5 (7.0-10.1) | 0.0005 |
| From young adulthood to late adulthood | | | | | |
| Stable non-obesity | 76.3 (73.6-78.9) | 68.3 (65.3-71.3) | 64.5 (61.4-67.6) | 60.2 (57.3-63.1) | <.0001 |
| Weight gain | 20.5 (18.1-22.9) | 29.1 (26.3-32.0) | 32.8 (29.9-35.7) | 37.2 (34.4-40.0) | <.0001 |
| Stable obesity | 2.0 (1.2-2.9) | 1.5 (0.8-2.2) | 2.2 (1.4-3.0) | 2.2 (1.3-3.2) | 0.5863 |
| Weight loss | 1.3 (0.7-1.8) | 1.1 (0.6-1.6) | 0.5 (0.2-0.8) | 0.4 (0.1-0.6) | 0.0005 |

^a^*P* value for trend was adjusted for age, sex, and race/ethnicity;

^b^*P* value for trend was adjusted for age and race/ethnicity.
